# Differentiated adaptative genetic architecture and language-related demographical history in South China inferred from 619 genomes from 56 populations

**DOI:** 10.1186/s12915-024-01854-9

**Published:** 2024-03-06

**Authors:** Qiuxia Sun, Mengge Wang, Tao Lu, Shuhan Duan, Yan Liu, Jing Chen, Zhiyong Wang, Yuntao Sun, Xiangping Li, Shaomei Wang, Liuyi Lu, Liping Hu, Libing Yun, Junbao Yang, Jiangwei Yan, Shengjie Nie, Yanfeng Zhu, Gang Chen, Chuan-Chao Wang, Chao Liu, Guanglin He, Renkuan Tang

**Affiliations:** 1 Department of Forensic Medicine, College of Basic Medicine, Chongqing Medical University, Chongqing, 400331 China; 2 Institute of Rare Diseases, West China Hospital of Sichuan University, Sichuan University, Chengdu, 610000 China; 3 School of Basic Medical Sciences, North Sichuan Medical College, Nanchong, 637100 China; 4 School of Clinical Medical Sciences, North Sichuan Medical College, Nanchong, 637100 China; 5 School of Forensic Medicine, Shanxi Medical University, Jinzhong, 030001 China; 6 School of Forensic Medicine, Kunming Medical University, Kunming, 650500 China; 7 West China School of Basic Science & Forensic Medicine, Sichuan University, Chengdu, 610041 China; 8 Department of Public Health, Chengdu Medical College, Chengdu, 610500 China; 9 Faculty of Forensic Medicine, Zhongshan School of Medicine, Sun Yat-Sen University, Guangzhou, 510275 China; 10 Guangzhou Forensic Science Institute, Guangzhou, 510055 China; 11 Anti-Drug Technology Center of Guangdong Province, Guangzhou, 510230 China; 12 Hunan Key Lab of Bioinformatics, School of Computer Science and Engineering, Central South University, Changsha, 410075 China; 13 State Key Laboratory of Cellular Stress Biology, National Institute for Data Science in Health and Medicine, School of Life Sciences, Xiamen University, Xiamen, 361005 Fujian China; 14 Center for Archaeological Science, Sichuan University, Chengdu, 610000 China

**Keywords:** Demographical history, Biological adaptation, Genetic structure, Genomic diversity, Han Chinese

## Abstract

**Background:**

The underrepresentation of human genomic resources from Southern Chinese populations limited their health equality in the precision medicine era and complete understanding of their genetic formation, admixture, and adaptive features. Besides, linguistical and genetic evidence supported the controversial hypothesis of their origin processes. One hotspot case was from the Chinese Guangxi Pinghua Han people (GPH), whose language was significantly similar to Southern Chinese dialects but whose uniparental gene pool was phylogenetically associated with the indigenous Tai-Kadai (TK) people. Here, we analyzed genome-wide SNP data in 619 people from four language families and 56 geographically different populations, in which 261 people from 21 geographically distinct populations were first reported here.

**Results:**

We identified significant population stratification among ethnolinguistically diverse Guangxi populations, suggesting their differentiated genetic origin and admixture processes. GPH shared more alleles related to Zhuang than Southern Han Chinese but received more northern ancestry relative to Zhuang. Admixture models and estimates of genetic distances showed that GPH had a close genetic relationship with geographically close TK compared to Northern Han Chinese, supporting their admixture origin hypothesis. Further admixture time and demographic history reconstruction supported GPH was formed via admixture between Northern Han Chinese and Southern TK people. We identified robust signatures associated with lipid metabolisms, such as fatty acid desaturases (FADS) and medically relevant loci associated with Mendelian disorder (GJB2) and complex diseases. We also explored the shared and unique selection signatures of ethnically different but linguistically related Guangxi lineages and found some shared signals related to immune and malaria resistance.

**Conclusions:**

Our genetic analysis illuminated the language-related fine-scale genetic structure and provided robust genetic evidence to support the admixture hypothesis that can explain the pattern of observed genetic diversity and formation of GPH. This work presented one comprehensive analysis focused on the population history and demographical adaptative process, which provided genetic evidence for personal health management and disease risk prediction models from Guangxi people. Further large-scale whole-genome sequencing projects would provide the entire landscape of southern Chinese genomic diversity and their contributions to human health and disease traits.

**Supplementary Information:**

The online version contains supplementary material available at 10.1186/s12915-024-01854-9.

## Background

Human genetic studies focused on understanding the genetic basis of the complex traits or clinical disorders were mainly conducted in European-related populations, such as UK10K [1] and FinnGen genomic resources [2]. Recent genome-wide studies regarding human genetic diversity, population relationships, and fine-scale genetic structures were also primarily focused on Europeans [3, 4], and other geographically diverse global populations were vastly underrepresented in human genetic research. Eurocentric biases will exacerbate disparities in the application of genome-wide association studies (GWAS) and clinical genome medicine. Besides, the insufficient genetic structure will introduce spurious associations when conducting GWAS analysis [3, 5]. Meanwhile, medical genetic studies have revealed various demographic histories and population structures influencing mapping for human diseases and phenotypes. Nevertheless, understanding the genetic architectures by which most groups impact associated diseases and traits remains elusive. Therefore, more studies should be conducted in these underrepresented populations to ameliorate these issues.

East Asia, one of the underrepresented groups, is home to rich human genetic, ethnic, and linguistic diversity, with China as the most populous country. There are complex patterns of genetic interactions between East Asia and adjacent regions across various time points, such as gene flow from or into Southeast Asia and Siberia [6–8]. Previous genetic studies revealed the genetic relationship among present-day East Asians was intricate, and the region’s complex demography remained poorly interrogated [9, 10]. Furthermore, genetic introgression from archaic groups (including Denisovan and Neanderthal) was also found in East Asians and other groups after humans out of Africa, facilitating the adaptation of the modern humans associated with multiple phenotypes, including immune function, altitude adaptation, and metabolism [11, 12]. Compared with large-scale ongoing genomic projects of the Trans-Omics for Precision Medicine (TOPMed) program [13] and the Genome Aggregation Database (gnomAD) [14] conducted in Europe and America, the population-specific genomic database from East Asia, particularly in China, is largely uncharacterized.

Recently, multiple large-scale whole-genome sequencing (WGS) analyses have been conducted, including The STROMICS genome study [15], Non-Invasive Prenatal Testing (NIPT) [16], 1001 Tibetan genomes project [17], China Metabolic Analytics Project (ChinaMap) [18], Nyuwa genomic resource [19], and the Westlake BioBank for Chinese (WBBC) [20], which promote a better understanding of the genetic background of Chinese populations and their medical relevance. To provide a high utility of genomic medicine in China, at least four high-resolution haplotype-resolved reference panels were constructed for Chinese populations recently, which can directly facilitate the advancement of precision medicine in China [21]. A series of Han-based large-scale genomic studies filled the gap of missing diversity in human genomes, including the Guangzhou birth cohort [22], ChinaMap [18], Nyuwa [19], WBBC [20], China Kadoorie Biobank (CKB) [21], and Chinese non-small cell lung cancer (NSCLC) cohort [23], which increased our understanding of genetic knowledge of East Asian-specific variations and association with traits. These reported Chinese genomic studies aggravated the missing genetic diversity of non-Han populations in evolutionary and genomic studies, which introduced the Han bias in genetic studies. Our previous demographical models have identified geography/language-related population substructures in China [24–27], respectively related to Altaic, Sino-Tibetan [Sinitic and Tibeto-Burman (TB)], Hmong-Mien (HM), Tai-Kadai (TK), Austronesian (AN), and Austroasiatic (AA) language families from North, Central, and South China. More work focused on the interaction of geographically high-coverage incoming and indigenous populations should be conducted to illuminate the influence of admixture between ethnic minorities and Han Chinese on human evolutionary adaptation and individual health management, which remained unknown and should be explored. Han Chinese is the largest community in China and the world, accounting for ~ 20% of the global human population and ~ 90% of the Chinese. There were also complicated gene flows between Han and surrounding ethnic communities in China [28, 29]. The one-dimensional “north-to-south” genetic cline was observed in Han populations associated with geographic distributions and historically documented migration events [30]. Several earlier analyses also reported evidence of a “north-to-south” cline among the geographically diverse Han populations, including Northern/Southern/Southeast/Southwest/Central Han [29]. Cao et al. classified the Han Chinese populations into seven clusters: Northwest Han, North Han, East Han, Central Han, Southeast Han, South Han, and Lingnan Han [18], whereas Cong et al. divided Han populations into three categories: North Han, Central Han, and Lingnan Han [20]. The presence of Lingnan Han in both two classifications indicates that Lingnan Han (including that in Guangxi Province) is important but ignored in the early genetic studies, while the fine-scale genetic structure, adaptation, and medical relevance of Lingnan Han remain largely unexplored.

Guangxi is a Southern region of China, bordering Guizhou and Hunan in the Northwest and Northeast, respectively, Guangdong in the East, Yunnan in the West, and Vietnam in the Southwest. Of these, Guizhou, Hunan, and Guangdong are provinces in China, while Vietnam is a neighboring country of China. This basined region is situated in the transitional area between the Southwest margin of the Yungui Plateau and the Western of two vast hills with genetically distinct ethnic populations. With a population of up to 50,126,804 [31] and 12 ethnic minority groups, Zhuang is the primary component, followed by Han and HM-speaking Miao. Pinghua is the primary language for communication between Han Chinese and Southern ethnic minorities, related to the earliest Han Chinese immigrants into Guangxi [32]. As one of the ten significant dialects (Pinghua, Jing, Cantonese, Wu, Hui, Xiang, Hakka, Gan, Min, and Mandarin) in China, the linguistic characteristics of Pinghua distinguish them from the other Guangxi dialects (Hakka and Mandarin) [33]. There is a recurrent interest in identifying the relationship between Pinghua and Cantonese for Chinese linguistics, and some linguistics supposed that Pinghua is considerably distinct from Cantonese [34]. The Pinghua dialect is divided into two subgroups, the Northern Pinghua and Southern Pinghua, and genetic studies targeting GPH have assessed the existence of differentiations between Northern and Southern Pinghua populations [35]. A previous study focused on mitochondrial and Y-chromosomal lineages suggested that GPH was an exception of the Han Chinese branches but the descendant of the Southern indigenous people, which assimilated by the Han groups in terms of language, culture, and self-identification [36]. Uniparental evidence supported the indigenous origin hypothesis of GPH and stated that cultural diffusion participated in the formation of GPH [36]. However, genome-wide studies for reconstructing the origin of GPH are missing until now. Our previous genome-wide evolutionary studies from Southeastern coastal Fujian Han and Tanka have reported extensive admixture signatures between Northern Han and Southern indigenous (HM, TK, AA, and AN speakers), providing genomic evidence for the admixture hypothesis for their genetic origin [37, 38]. Other genetic studies focused on the single ethnic group have explored their genetic relationship but ignored fine-scale genetic structure based on the shared haplotypes and their potentially differentiated biological adaptative features [39, 40]. Therefore, compared with other well-studied Southern Han Chinese in South China, the GPH, who speaks one of the unique Chinese dialects, is under-represented. It is also unknown whether the observed adaptive evolution of Southern Chinese populations can be applied to any other Southern Han Chinese, such as GPH. Thus, a comprehensive population genetic study is imperative to explore the genetic origin, diversity, migration, biological adaptation, and admixture processes of modern GPH and the genetic relationships with surrounding ethnolinguistically different populations.

Consequently, we presented a comprehensive genome-wide study of 619 genomes from 56 ethnolinguistically Guangxi people related to HM-speaking Miao and Yao, TK-speaking Zhuang and Sui, AA-speaking Jing, and Sinitic-speaking Han, which included 261 GPH genomes first reported here. We merged our data with the publicly available modern and ancient genetic data to explore the population structure, demographic history, and local adaptations of the GPH. We provided robust genomic evidence for supporting the admixture hypothesis for the origin of GPH. Our admixture and demographical models identified geography/linguistic-related genetic structure, which suggested the importance of including more ancestrally diverse populations in human genomic studies. Comprehensive biological adaptation analysis based on the differentiated shared alleles and haplotypes identified many top selection signatures and highly differentiated loci associated with lipid metabolism and medical-related traits. The reconstructed demographic history and adaptive genetic features of the GPH and their neighbors are significant for understanding the evolution of modern humans and equivalent precision medicine in a larger proportion of worldwide groups.

## Results

### Differentiated genetic affinities of GPH and their neighbors

**Fig. 1:**
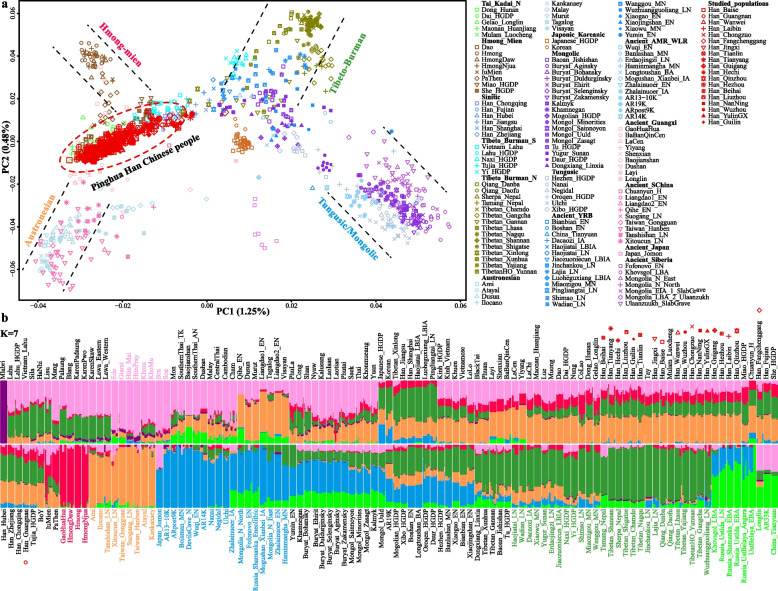
General population structure inferred from principal component analysis and model-based ADMIXTURE. **a** Patterns of genetic relationship among 93 ethnolinguistically distinct modern East Asians and 58 ancient populations, in which ancient people were projected into the essential background of two top components extracted from modern genetic variations. Populations from different language families or archeologically different groups were color-coded with different colors and different shapes, presenting different populations within one language family or archeological group. **b** Individual or population ancestry components among 210 ancient and modern Eastern Eurasian groups were inferred based on the ADMIXTURE with seven predefined ancestral sources. The model with *K* = 7 possessed the lowest cross-validation error

We newly generated genome-wide genotype dataset of 261 individuals, including 236 Sinitic-speaking individuals from 19 populations across Guangxi Province and 25 Sinitic speakers from two populations in Yunnan Province, combined them with previously reported Guangxi populations, and formed a basal studied dataset including 619 Guangxi genomes (Additional file 1: Fig. S1). We merged it with published population data with different single-nucleotide polymorphism (SNP) densities and formed four datasets. We merged it with previously reported Affymetrix genotyping data from ethnolinguistically diverse Chinese populations (Mongolian, Han, Tujia, Miao, Sui, Jing, Zhuang, and Li) [26, 39–43] and formed the high-density SNP panel dataset (465,941 SNPs). Populations from the Human Genome Diversity Project (HGDP) and Oceania genomic resources were included in our high-density merged HGDP-O dataset [12, 44]. The Human Origin (HO) dataset and the 1240 K dataset from Allen Ancient DNA Resource (AADR) [45] were merged to form the low-density merged HO and middle-density merged 1240 K datasets, to explore the relationship with different ancient genomes especially ancient individuals in Guangxi [46]. To characterize the genetic profile of GPH, we first performed principal component analysis (PCA) to explore the genetic affinities between GPH and other references in modern and ancient Eastern Asian contexts (Fig. 1a). The genetic difference generated from the first component (PC1: 1.25%) has differentiated HM and AN speakers in South China from TB and Tungusic/Mongolic people in North China. The second component (PC2: 0.48%) has distinguished HM and TB speakers from AN and Tungusic/Mongolic people. PCA based on the merged HO dataset demonstrated that GPH was adjacent to TB at one end and AN at the other, overlapping with TK groups and part of HM-, Sinitic-, and TB-related clines. We further projected ancient samples into the background of modern East Asians to explore the genetic affinity between target populations and ancient East Asians. Our studied populations showed a close genetic similarity with ancient Guangxi individuals and were located between ancient Northern millet farmers from the Yellow River Basin (YRB) and ancient Southern East Asian (ASEA)-related populations. The observed patterns suggested that GPH’s ancestries were possibly related to the descendants of these two groups and their different demographic interactions compared to other Han populations.

**Fig. 2:**
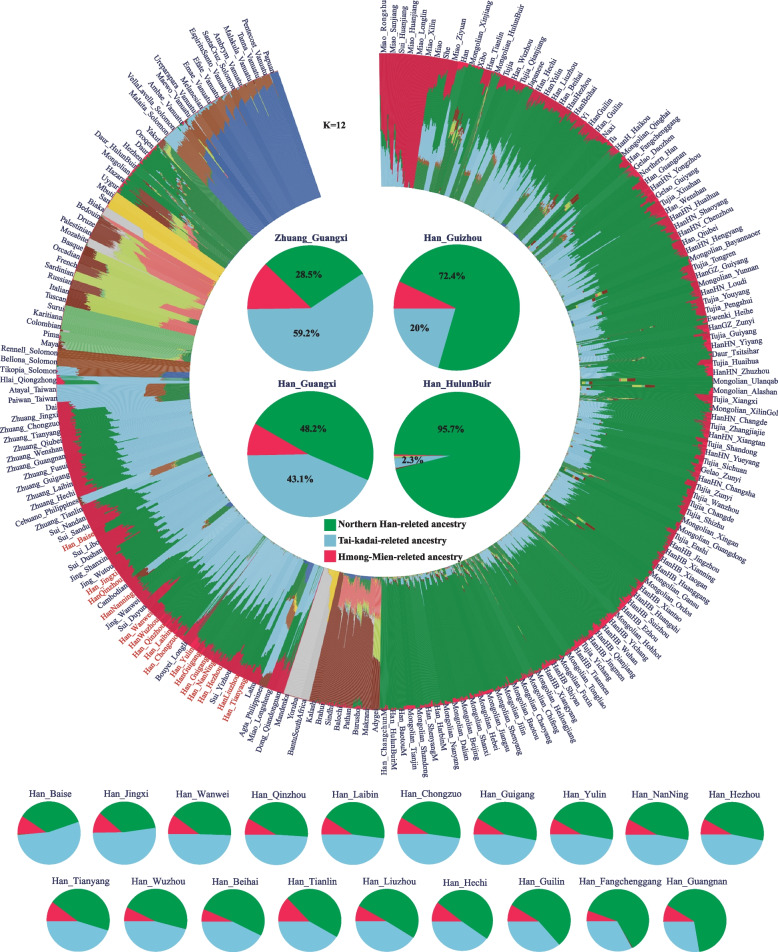
Unsupervised ADMIXTURE results with *K* = 12. Admixture analysis of GPH shown with red, green and light blue components maximized in Hmong-Mien-, Northern Han-, and Tai-Kadai (TK)-related groups, respectively. Four groups (including Northern Han, Southern Han, Southern TK, and GPH groups) were selected individually to present their specific ancestral composition and proportions. The 19 Guangxi groups were arranged from left to right as the Northern Han-related components increased

The well-fitted ADMIXTURE model based on the merged HO dataset revealed that the newly studied GPH harbored the highest proportion of AN-related ancestry (ancestral component colored as orange) and a certain proportion of TB- (green), HM- (red), and AA-related (pink) ancestries (Fig. 1b). The ADMIXTURE-based admixture model showed that GPH derived ancestries from Northern and Southern ancestral sources, which was in line with the result of PCA, supporting the view that GPH derived from these ancient Northern East Asians (ANEAs) and ASEAs. Furthermore, the complex genetic relationship has attracted our interest in exploring the genetic contribution of Northern Han to GPH. We performed ADMIXTURE in the context of the merged HGDP-O dataset, which showed that there were most likely three ancestral populations that contributed to GPH (Fig. 2). The ADMIXTURE result indicated that GPH shared the majority ancestral makeup with Northern Han-related ancestry (ancestral component colored as green, proportion of 48.2%), followed by Zhuang- (43.1%) and Miao-related (8.7%) ancestry. The ancestral composition pattern of GPH was similar to that of Guangxi Zhuang (GXZ). To further explore the distribution patterns of the ancestral components related to Northern Han, we compared the ancestral proportion of geographically different Han and Southern indigenous groups, including Northern Han (Han_HulunBuir), Southern Han (Han_Guizhou), Southern ethnic minority (GXZ), and GPH. The Han-related component consistently decreased from North to South China, while the Zhuang-related component increased, consistent with the geographic location. Besides, we explored the similarities of geographically different GPH populations, and no statistically significant population stratifications or correlations with longitude or latitude were observed (*R*^2^ < 0.5).

**Fig. 3:**
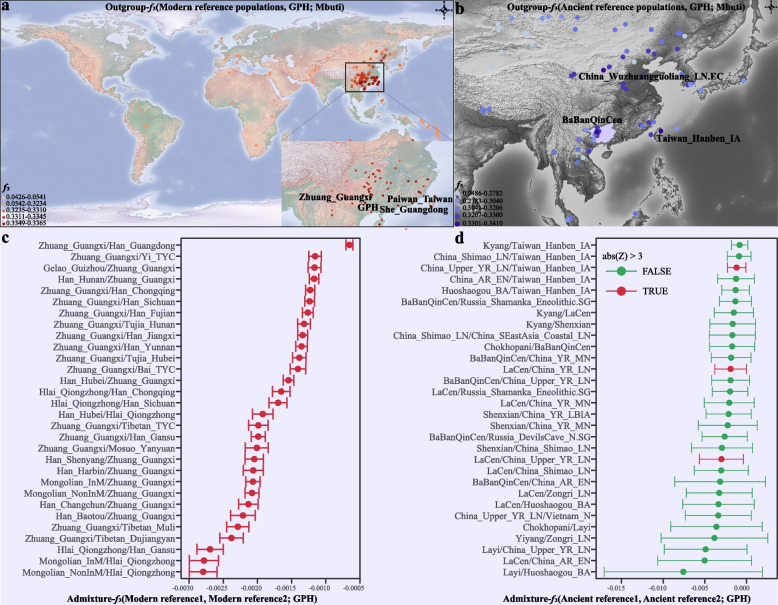
Outgroup-*f*_*3*_ statistics and admixture-*f*_*3*_ statistics for GPHs. **a**, **b** Outgroup-*f*_3_ statistics based on the merged HGDP-O data and the merged 1240 K data to explore the geographic distribution pattern of genetic drift shared between modern groups, ancient populations, and GPH, respectively. Different colors represent different levels of *f*_*3*_ values. See Additional file 1: Figs. S2b and S4 for more details. **c**, **d** We conducted admixture-*f*_3_ statistics of the form *f*_3_ (modern reference population1/ancient reference population1, modern reference population2/ancient reference population2; GPH), and the top 30 were respectively shown in **c** and **d** after filtering. Red represents *Z* values that are significant, and green represents *Z* values that are not significant

GPH falls in the genetic variability between Northern and Southern East Asians (NEA and SEA) in several analyses and demonstrates a high genetic affinity to Southern ethnic minorities. We calculated the pairwise Fst values to examine the genetic relationship between GPH and surrounding populations (Additional file 1: Fig. S2a). The overall genetic makeup of GPH was closest to Han_Haikou (Fst = 0.0010), followed by surrounding Han and ethnic minorities, including GXZ, Han_Hunan, and Han_Guizhou (Fst = 0.0013–0.0017). The estimated affinity based on the pairwise genetic distances demonstrated that GPH had a closer genetic relationship with Han and ethnic minorities of geographically adjacent populations from South China than with Northern Han. We further confirmed the genetic affinity between GPH and geographically close populations by using outgroup-*f*_3_ statistics as *f*_3_ (modern reference populations, GPH; Mbuti), which detected the amount of shared genetic drift between modern worldwide groups and newly studied populations. GPH generally had the most significant outgroup-*f*_3_ values and the most shared genetic drift with Southern ethnic minorities, such as She, Paiwan, Zhuang, and other Southern groups (Fig. 3a; Additional file 1: Fig. S2b). Our previous analysis of Fst and outgroup-*f*_3_ statistics suggested a more robust genetic affinity between GPH and surrounding populations (Fig. 3a; Additional file 1: Fig. S2a-b). We further confirmed the genetic links by calculating the shared IBD (identity by descent) among East Asian populations using Refined IBD [47], which again showed frequent genetic exchanges between GPH and surrounding populations, especially ethnic minorities from Guangxi and Guizhou Provinces at a wide range of time, suggesting that GPH received genetic influence from geographically close populations (Additional file 1: Fig. S3a-f). Next, the genetic affinity between GPH and 30 ancient Chinese populations was also quantified by calculating outgroup-*f*_*3*_ statistics in the form of *f*_3_ (ancient reference populations, GPH; Mbuti) and found that GPH shared the most alleles with Iron Age and historic ASEAs, including Taiwan_Hanben_IA, BaBanQinChen, and LaCen (Fig. 3b; Additional file 1: Fig. S4). These observations on outgroup-*f*_3_ statistics are also confirmed by the *f*_4_ statistics in the form of *f*_4_ (ancient reference population1, ancient reference population2; GPH, Mbuti) (Additional file 1: Fig. S5). As described above, GPH possessed genetic similarities to modern and ancient Southern East Asians.

### Admixture scenarios and gene flows

In order to assess the admixture scenarios revealed by the ADMIXTURE analysis, we investigated whether it could recover the Southern and Northern genetic ancestries by simulating GPH as a mixture of all available pairs of sources. We performed admixture-*f*_3_ statistics using modern plausible ancestral sources included in the merged HGDP-O dataset to explore the potential ancestry groups of GPH. Most of the tests with GXZ and Northern Han groups as source pairs are statistically significantly negative (*Z* scores < − 3), indicating that the gene pool of GPH can be modeled as the result of the north-to-south admixture model (Fig. 3c), which was consistent with the observed mixed pattern in the fitted ADMIXTURE models. We also observed that the combination of Southern Han and Southern ethnic minorities had significant negative values, suggesting that GPH possessed additional genetic material compared with the other Southern Han. To test whether source pairs of ANEAs and ASEAs can be used to fit the observed genetic diversity of GPH, we then conducted the admixture-*f*_*3*_ analysis using the potential ancestral source groups included in the merged 1240 K dataset. We observed statistically significant negative *Z* scores in *f*_*3*_ (ancient reference population1, ancient reference population2; GPH), especially the LaCen/Taiwan_Hanben_IA and China_Upper_YR_LN pairs, suggesting that GPH was an admixed population and could be simulated as an admixture of these groups (Fig. 3d). In addition, a few pairs of two ancient people from South China could also be used as possible ancestral sources of GPH.

Furthermore, we performed a series of *f*_*4*_ statistics in different forms. To assess the degree of genetic homogeneity within GPH groups, we carried out symmetrical *f*_4_ (GPH1, GPH2; reference populations, Mbuti) based on the merged HO dataset. A significant positive-*f*_4_ value indicated more allele sharing between reference populations and GPH1, while a significant negative-*f*_4_ value indicated more allele sharing between reference populations and GPH2. Most results with non-significant *f*_4_ value (|*Z*|≤ 3) were observed, which indicated the relative genetic homogeneity in GPH from different Guangxi prefecture-level cities. However, a few results of symmetrical *f*_4_ (GPH1, GPH2; reference populations, Mbuti) with significant *f*_4_ value (|*Z*|> 3) were also identified here, such as Northwest and Northeast GPH (Baise and Yulin) showed more SEA-related ancestry, including AN-, TK-, and AA-related ancestry when compared with Southwestern GPH (Fangchenggang) (Additional file 2: Table S1). The result showed that several GPH people had genetic heterogeneity and contained different genetic admixtures and evolutionary histories but generally genetic homogeneity within GPH. For the remaining analyses, GPH were merged into a single cluster based on their minimal genetic heterogeneity.

To quantify the genetic heterogeneity between GPH and ethnically and geographically different people, we focused on the differences between Southern ethnic minorities and Hans by calculating *f*_4_ statistics in the form of *f*_4_ (GPH, Guangxi ethnic minorities/Northern Han/Southern Han; reference populations, Mbuti). GPH shared more SEA-related alleles than Northern Han (Han_HulunBuir) (Additional file 1: Fig. S6a). We observed statistically significant *f*_4_ values, indicating the differentiated demographical history of ethnolinguistically different Guangxi people, and GPH shared more NEA-related alleles when compared to Southern ethnic minorities, including Hlai, Jing, and Zhuang (Additional file 1: Fig. S6b-d). The estimated positive values in *f*_4_ (GPH, Han_Haikou; reference populations, Mbuti) further suggested that GPH received additional genetic influence from Southern ethnic minorities than Southern Han, confirming the admixture signatures inferred from the admixture-*f*_*3*_ statistics (Additional file 1: Fig. S7a). The genetic discrepancy between GPH and Guangxi Miao, Sui, and Yao groups was also confirmed via statistically significant *f*_4_ (GPH, Guangxi ethnic minorities; reference populations, Mbuti) (Additional file 1: Fig. S7b-d). We then examined the genetic cladality between our studied populations and modern Chinese people using an individual-based qpWave analysis, and we observed significant statistical differences between GPH and other Han Chinese from North and South China. The potential reason could be that GPH harbored more indigenous SEA-related components than other Hans, which mainly contributed from GXZ (Additional file 2: Table S2). Genetic heterogeneity also existed between GPH and GXZ populations. Besides, non-significant statistical values were observed between GPH and some geographically close groups (Dongs, Jings, Shes, Yaos, and Miaos). As stated above, GPH was genetically divergent from both Han Chinese groups and Southern ethnic minorities, further supporting the formation of the north-to-south admixture. We found two potential ancestral proxies of GPH based on the resulting admixture-*f*_3_ statistics, including the Northern Han or ANEA and the other GXZ or ASEA. We then performed *f*_4_ (reference populations, GPH; Northern Han, Mbuti) and used all publicly available East Asian populations as the reference populations (Additional file 2: Table S3). Statistically significant negative *f*_4_ values indicated that GPH showed closer genetic connections with Northern Han than other East Asian groups. Furthermore, to test whether GPH directly from the Northern Han was plausible, we computed the *f*_4_ (Northern Han, GPH; reference populations, Mbuti) to determine whether newly studied populations and Northern Han form a robust clade, where reference populations were 133 worldwide groups. However, we observed significant negative *f*_4_ values when SEAs related to Hlai, Zhuang, and Sui were used as the reference groups, indicating that GPH obtained additional gene flow from SEA compared to their Northern ancestral proximity (Additional file 2: Table S4). These observations were also supported and confirmed by the admixture-*f*_3_ statistics. Generally, affinity statistics showed GPH and GXZ had significant genetic heterogeneity and the former shared more alleles with Northern Han relative to GXZ, which provided the clues supporting both millet and rice farmers participating in the formation of GPH. To test our hypothesis, we then conducted *f*_4_ (ancient reference populations, GPH; ASEA, Mbuti) to test the affinity with ASEA sources, where other ancient East Asians represented ancient reference populations. We observed significant negative values, which suggested ASEA shared more alleles with GPH than other reference groups and is consistent with the expectation in the hypothesis status (Additional file 1: Fig. S8a). To identify signals of additional genetic materials of GPH, we selected Taiwan_Hanben_IA, LaCen, and BaBanQinCen as the Southern ancestral source and performed *f*_4_ (ASEA, GPH; ancient reference populations, Mbuti). We identified several Northern ancestral sources, particularly China_Upper_YR_LN, which possessed the most significant negative value compared to Taiwan_Hanben_IA (Additional file 1: Fig. S8b), suggesting their additional gene flow into GPH. As stated above, these results combined the admixture signatures identified in the admixture *f*_3_ statistics, suggesting that two ancient ancestries from North and South China participated in the formation of GPH, especially the combination of ancient people from YRB in the late Neolithic period and ancient groups from Taiwan Hanben in the Iron Age, which was in line with the admixture-*f*_3_ statistics models.

Finally, we also estimated the ROH (runs of homozygosity) to measure the recent inbreeding of GPH and other East Asians under the merged HO dataset using PLINK v.1.90 [48], illustrating that Miao_Guangxi have many long ROH segments within populations related to other groups and GPH shared an approximately close number of ROH segments with other Hans and surrounding groups (Additional file 1: Fig. S9a-e). We observed the same pattern of ROH segments in three different levels in Miao_Guangxi. One possible explanation could be that Miao_Guangxi has experienced more consanguineous marriages compared to their surrounding populations, and there were different situations of consanguineous marriage among different groups in Guangxi Province.

### Graph-based complex evolutionary models

**Fig. 4:**
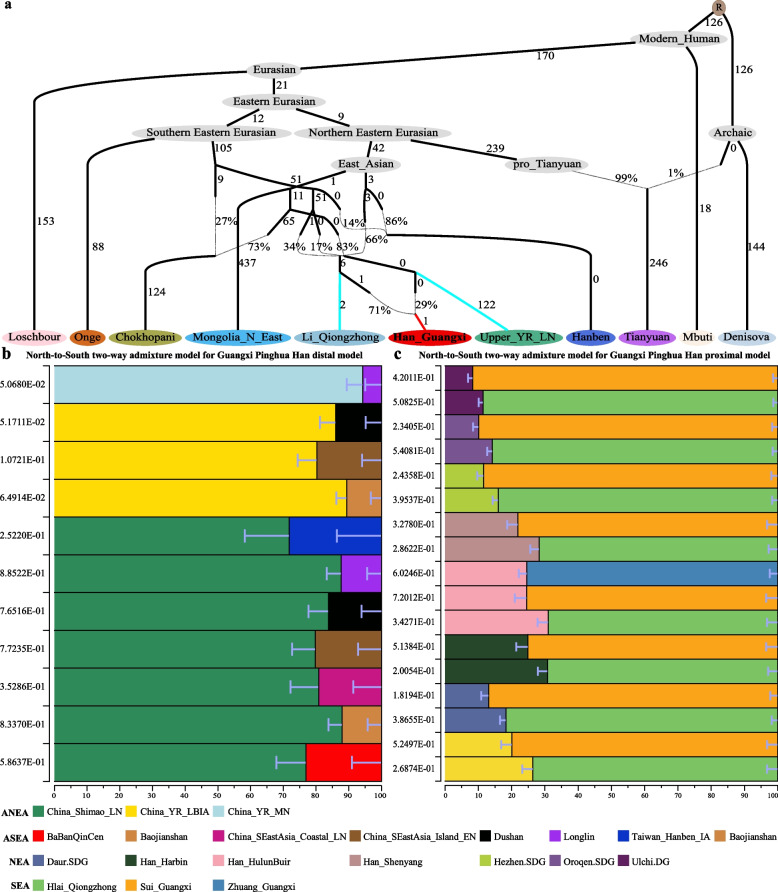
Genetic ancestry modeling for potential sources across newly reported genetic groups. **a** Evolutionary history with five admixture events constructed by qpGraph. The selected qpGraph-based phylogenetic topology fitted the best-worst *Z* scores below 3 (*Z* = − 2.674). Percentages on the dashed lines represent the admixture proportions of the two ancestral groups. Numbers on solid lines show 1000 times genetic drift. Admixture graph fitting GPH as an admixture of ancestries associated with Li_Qiongzhong and Upper_YR_LN. **b** Working distal qpAdm models with distinct ancestral sources. GPH show high levels of YRB ancestry. The error bar indicates the stand errors of predicted proportions of ancestors obtained from qpAdm. **c** The admixture proportions of proximal qpAdm models for GPH. Each bar represents ancestry proportions of the listed subgroups for GPH. ANEA, ancient Northern East Asian; ASEA, ancient Southern East Asian; NEA, Northern East Asian; SEA, Southern East Asian

To construct the admixture graph, including gene flow events and population split between the diverged human populations, we performed TreeMix-based phylogenetic tree reconstructions. Phylogenetic relationships within 29 modern populations without admixture events portrayed two lineages following the geographic distribution. One was the lineage of Northern Han, and the other was Southern ethnic minorities. Our target population was located on the Southern indigenous genetic lineage, which closely clustered with Han_Haikou, followed by surrounding ethnic minorities (Additional file 1: Fig. S10a). However, with the increasing number of gene flow events, we identified gene flow from the Tujia_Guizhou into GPH with admixture events, which showed frequent genetic exchanges among surrounding populations. Generally, GPH showed a strong affinity to geographically close populations, including ethnic minorities of Guangxi and Guizhou Provinces and placed in the Southern indigenous lineage (Additional file 1: Fig. S10b-d). Additionally, results based on the shared IBD, Fst, outgroup-*f*_3_ statistics, and *f*_4_ statistics among individual-level or population-level groups also confirmed the genetic affinity within the geographically adjacent ethnic minorities. Furthermore, we modeled the relationship between GPH and Southern ethnic minorities using a graph-based qpGraph method. We observed that the Li_Qiongzhong-related and Upper_YellowRiver_LN-related lineages contributed to modern GPH with different proportions (*Z* = − 2.674). GPH was fitted with 29% ancestry related to Upper_YellowRiver_LN and the primary (71%) ancestry from Li-Qiongzhong, which revealed the potential genetic pattern of GPH that resulted from the expansion of the ancient YRB-related ancestries to South China (Fig. 4a). Admixture modeling confirms the contribution of ANEA and SEA among the GPH. To quantify the fine-scale ancestral proportion of different ancestry sources in studied populations, we used qpAdm modeling analysis. We considered that the qpAdm model is rejected if the *p* value < 0.05 and admixture proportions exceed the bound of 0–1 and the stand error is below zero [49]. To better define the genetic link between Neolithic to Iron Age populations and GPH, we used Neolithic to Iron Age populations as the distal sources for the qpAdm models and observed GPH could be modeled using YRB populations (71.8 to 94.3%) with additional ancestry from Guangxi and Fujian sources (5.7–28.2%) (Fig. 4b). Additionally, models using more contemporaneous potential ancestral surrogates portray GPH as a mixture of two major ancestry sources that are descended from ancestry present in the NEA/ANEA and Southern ethnic minorities (Fig. 4c). The genetic heterogeneity between GPH and the ancestry sources was examined using qpWave analysis (Additional file 2: Tables S5-S6). In addition, the three-way admixture model of Han-Atayal-GXZ (0.264-0.052-0.684, respectively) could also provide a good fit for GPH’s admixture history (Additional file 2: Table S7). Taken together, we find two distinct and geographically structured ancestry sources contributed to the gene pools of GPH, with the ANEA/NEA population representing one of them. We refer to the other one as ASEA/SEA.

We further used the ALDER (Admixture-induced Linkage Disequilibrium for Evolutionary Relationship)-based method to infer the date of admixture events between two ancestral populations with a generation time of 29 years [50]. Our observed result showed complex genetic admixture processes between NEA and SEA in studied populations, such as the evidence of admixture with standard deviation of 24.90 and 13.57 for GPH 77.32 generations (985.38 BCE–485.82 CE) in the Han_Harbin-GXZ model and 70.84 generations (468.89 BCE–318.17 CE) for the Han_Changchun-GXZ simulation, respectively (Additional file 2: Table S8). Linkage-based admixture time estimation suggested that our target groups can be modeled as Northern Han and GXZ admixture results in a wide range of time. To further identify, date, and describe the fine-scale admixture events and get more detailed information on the demographic history of GPH, we conducted the fastGLOBETROTTER analysis and used 14 genetically different populations (including 11 populations from East Asia, two populations from Europe and one population from South Asia) as surrogates for the admixture sources and employed Han_Harbin as the possible Northern donor and Cambodian as the Southern source (Additional file 1: Fig. S11a-b). In the provided output results, the admixture conclusion was “one date” at around 986 years ago (34 generations with a generation time of 29 years). Of the two sources contributing to the GPH, one was inferred to contribute 46% of the total admixture proportion and most genetically similar to the Cambodian groups. Analogously, the other source was inferred to contribute 54% and most genetically similar to Han_Harbin. This also supported the mixed north–south model as confirmed by several analysis.

### Detailed demographic history and fine-scale genetic structure of GPH

**Fig. 5:**
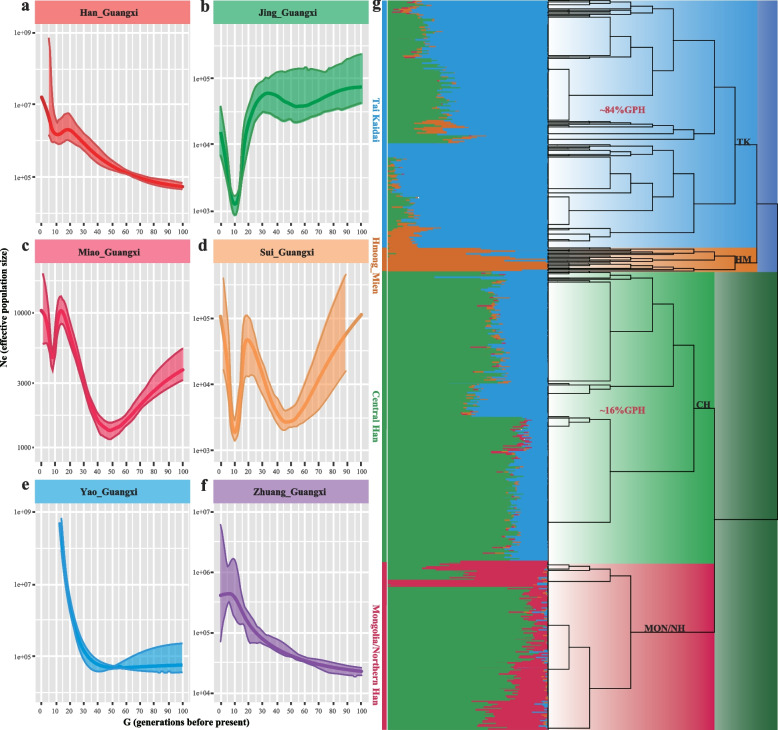
Estimated recent effective population size and fine-scale population structure. **a**–**f** Effective population size (Ne) inferred by IBDNe, using 619 individuals from 6 ethnolinguistic groups in Guangxi Province. **g** The maximum a posteriori (MAP) tree produced by fineSTRUCTURE exhibits clustering patterns among 30 different East Asian groups. These two percentages (~84% and ~16%) represent the proportion of GPH clustered with TK and Central Han-related branches, respectively. Adjacent to the tree is an ADMIXTURE plot for the same data with four predefined ancestral sources. The figure uses these abbreviations: TK, Tai-Kadai; HM, Hmong-Mien; CH, Central Han; Mon, Mongolia; NH, Northern Han; GPH, Guangxi Pinghua Han people

We used the merged HO dataset to infer demographic history and utilized IBDNe to infer GPH’s recent demographic history. Our observation found that Han from Guigang, Liuzhou, Guilin, and Qinzhou experienced a different demographic history, indicating that people from these four districts did not experience population bottlenecks in recent generations (Additional file 1: Fig. S12a-b). At the same time, most populations in Guangxi Province showed different degrees of population bottleneck around ten to fifty generations ago. Moreover, Han and Zhuang populations in Guangxi from previously published studies have also been used to infer demographic history. We found that Guilin and Qinzhou Hans did not experience a population bottleneck, which is consistent with our studied populations (Additional file 1: Fig. S12c). Similarly, a large majority of populations experienced a population bottleneck, in line with our newly generated populations, and GXZ has also occurred in a similar condition (Additional file 1: Fig. S12d). In addition, we also found that different language families in the same region have different demographic histories at the group level. We observed GPH, Jing, Miao, and Sui experienced a recent bottleneck around ten generations ago, while Yao and Zhuang did not (Fig. 5a–f). Furthermore, there was another bottleneck event only in Miao and Sui speakers around 50 generations ago, suggesting different demographic histories of linguistically diverse ethnic groups in the same region.

Genetic admixture analysis and demographical modeling based on the pattern of shared alleles between individuals can only capture the primary information of population history. The combination of high-density SNP data and advanced computation capacity enables the exploration of population history through the linked DNA segments [51]. We used the phased haplotype fragments of 1716 East Asian individuals to explore the fine-scale population structure. Patterns of shared ancestry inferred from haplotype data revealed the previously unknown population sub-cluster. Population dendrogram among GPH and other 29 East Asian groups based on the average chunk showed two main branches (Southern branch represented by Southern indigenous groups and Northern branch represented by Mongolian and Han Chinese populations) and finer-scale subbranches (Fig. 5g). We observed that GPH was mainly formed a clade from the Southern branch (~ 84%), and few GPH populations were located in the subbranches of the Central Han branch (~ 16%), suggesting that GPH had a closer genetic affinity with the Southern indigenous groups relative to Han populations and complex multifaceted admixture scenarios occurred between GPH and neighboring ethnolinguistic groups. Besides, we observed the genetic homogeneity within GPH from the pairwise coincidence matrix outputted by fineSTRUCTURE, which was in line with the results from the *f*_4_ statistics analysis (Additional file 1: Fig. S13).

### Highly differentiated genetics stratification within Guangxi populations

**Fig. 6:**
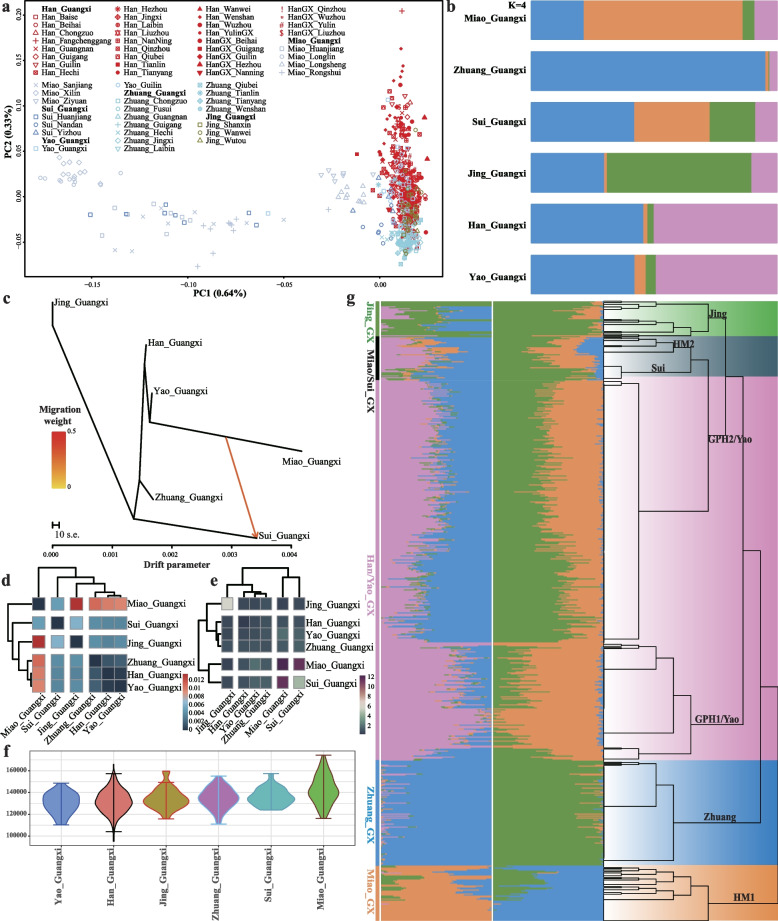
Demographic history of five indigenous communities and GPH in Guangxi. **a** 619 modern individuals covering six ethnic groups were projected onto the first two principal components. **b ***K* = 4 clustering analysis of six distinct cultural communities from the Guangxi Province using the ADMIXTURE method. **c** Inferred phylogenetic tree with one migration event. The migration arrow is assigned with appropriate weights. **d** The heatmap of pairwise Fst between GPH and five aboriginal groups of Guangxi. This shows the pattern of genetic closeness among six ethnic populations from the same geographic regions. **e** Heat map of the average length of paired IBD shared between populations. **f** Comparison of the long ROH (10–20 Mb) between populations. **g** Fine-scale population structure reconstructed based on the shared haplotypes. ADMIXTURE plot (*K* = 3 and *K* = 4) for 619 modern individuals, spanning six populations. Abbreviations of population names: Jing, Guangxi Jing; Sui, Guangxi Sui; Yao, Guangxi Yao; Zhuang, Guangxi Zhuang; GPH1, Guangxi Pinghua Han1; GPH2, Guangxi Pinghua Han2; HM1, Guangxi Miao1; HM2, Guangxi Miao2

To address the genetic history between GPH and other Guangxi indigenous populations, we performed analyses of 619 individuals comprising six ethnolinguistic groups to explore the fine-scale evolutionary process across Guangxi Province. The PC1 in Guangxi regional PCA separated the GPH, Jing, and Yao people from Miao and Sui speakers. Miao and Sui formed two sub-clusters, one of which was close to the other Guangxi group, indicating a complex structure within ethnolinguistically diverse Guangxi groups (Fig. 6a). We then used an unsupervised clustering method implemented in ADMIXTURE to investigate population structure. At *K* = 2, an orange component associated with Miao and a blue component dominant in Zhuang were observed (Additional file 1: Fig. S14a). For *K* = 3, a pink component enriched in GPH was found (Additional file 1: Fig. S14a); for *K* = 4, another green component appeared in Jing speakers (Fig. 6b). This revealed significant genetic differences among groups in the same region. TreeMix analysis with one gene flow from Miao to Sui showed genetic affinity between Guangxi Miao and Sui populations, consistent with PCA results (Fig. 6c). Besides, the six Guangxi populations were separated into three splits, and GPH had a close relationship with the Yao groups. The genetic relationship and population structure were further evaluated using a pairwise Fst heatmap (Fig. 6d). The total average degree of IBD sharing between population groups suggested that Miao was genetically closest to Sui and GPH and Yao was closest related to Zhuang (Fig. 6e), which is consistent with observations from PCA, TreeMix, and Fst. Demographic reconstructions further emphasized diverse profiles among Guangxi populations. The ROH showed a characteristic profile for the Sui and Miao groups, with ROHs longer than 10–20 Mb and the previous bin (5–15 Mb), which might suggest recent intermarriage happened within these two groups relative to the other four populations (Fig. 6f; Additional file 1: Fig. S14b). This was also supported by the distribution of the total number of ROH fragments (Additional file 1: Fig. S14c). This testified that high inbreeding in Sui and Miao individuals occurred and suggested a distinct demographic history for the other four groups. Such a genetic discrepancy among ethnically different Guangxi communities was also confirmed by the fineSTRUCTURE topology (Fig. 6g), where the Miao branch split earlier than the other Guangxi clusters. The Zhuang cluster was next split from other Guangxi groups before the subcluster in the rest of the populations. We observed a subcluster within GPH, which might be consistent with the genetic diversity between Northern and Southern GPH [35]. Yao scattered in two subclusters distributed in GPH, which suggested a close genetic affinity between these populations. Jing split from one of the subclusters of GPH and formed an independent clade before the Miao and Sui separated. The two clusters in Miao groups indicated a substructure within Miao groups, which was in line with the result of PCA. There were complex population structures and significant genetic discrepancies among individuals representing six diverse cultural communities from the same geographical region, which might reflect that GPH was not a Southern indigenous group.

### Uniparental genetic history suggested admixture models of GPH

To reconstruct the paternal and maternal structures of GPH, we comprehensively explored their non-recombining Y-chromosome (NRY) and mtDNA haplogroups. Four hundred seventy-four unrelated male individuals in Guangxi Province could be assigned into several terminal Y-chromosomal lineages. The constructed phylogenetic topology showed that O2a and O1 lineages were prevalent in GPH with the highest proportions and were regarded as the founder lineages of GPH (Additional file 1: Fig. S15). The O1b1a paternal lineage was dominant in GPH, which was also prevalent in SEA indigenous groups (AA and HM). The O2a2 was identified in GPH with a high frequency, which was consistent with the overall paternal profile of the other Han Chinese [52–54]. Besides, a few NEA lineages (N1a1, R1b1, R1a1, and Q1b1) were sporadically distributed in GPH, suggesting that our studied groups obtained gene flow from NEA. We also constructed the network relationship among 317 male individuals, suggesting the same haplogroup distribution pattern (Additional file 1: Fig. S16). As for the mtDNA haplogroup results, we constructed the network relationship among 619 female people. The phylogenic tree revealed that the main mtDNA haplogroup of GPH was primarily contributed by M* (M7b1a1 and M7c1) and followed by D4 lineages, which exhibited a pattern similar to Southern indigenous groups, especially TK-related populations. Other haplogroups (B4 and F1) were also identified in GPH with low frequency (Additional file 1: Fig. S17). The uniparental haplogroup distributions were consistent with previous observations [36].

### Shared and divergent adaptation signatures among ethnolinguistically diverse populations

**Fig. 7:**
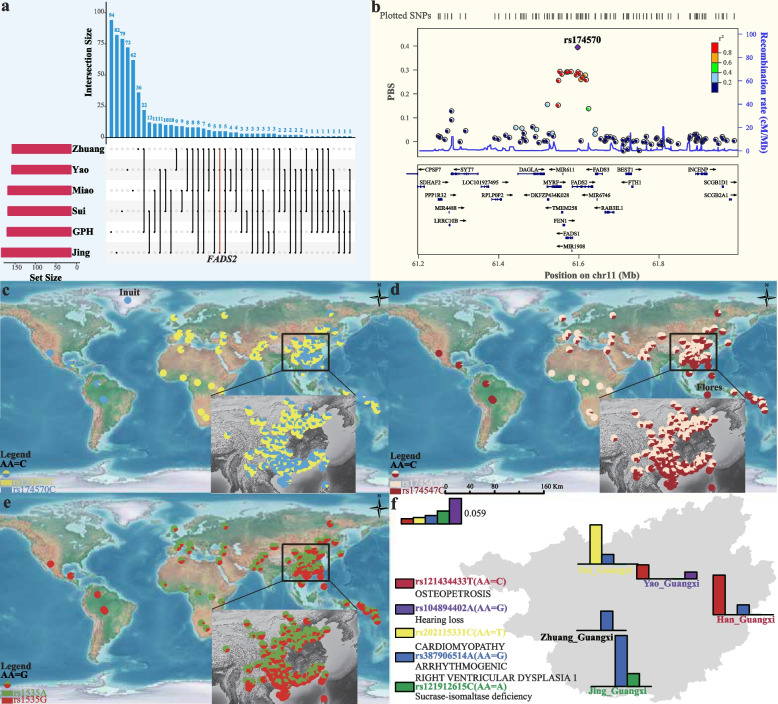
Positive selection and medical relevant variation in Guangxi. **a** The upset plot of population-specific natural selection signals using PBS in the form of (GPH/Zhuang/Miao/Yao/Sui/Jing_Guangxi-Han_Changchun-European). The upper bar represents the number of gene intersections between populations and population-specific genes. The bar graph on the left represents the original number of genes in the population. Black dots represent different populations, and black lines indicate gene sharing between two populations. *FADS2* was selected in the Zhuang, Yao, Sui, and Jing populations. **b** Regional plot for the top-ranked SNP rs174570 located in the *FADS2* gene, on chromosome 11. The concerning adaptive variant (rs174570) is marked, while other SNPs are colored based on the pairwise linkage disequilibrium with the target variant. **c**–**e** Geography distribution of genetic variants at rs174570, rs174547 and rs1535. These figures show the frequency distribution of *FADS2* (rs174570 C/T and rs1535 G/A) and *FADS1* (rs174547 C/T) and associated alleles based on the 10K_CPGDP database. The frequencies of Inuit and Flores were obtained from previously published studies. The ancestral allele (AA) was annotated using VEP [64]. **f** Distribution of medically relevant variation alleles across six ethnic cultural communities in Guangxi. In each group, the bar graphs indicate the corresponding proportions of the selected complex diseases or traits related alleles (the frequency of these five variants were zero in Miao_Guangxi group)

To identify the potential genes responsible for the population-specific adaptation of GPH, we looked for adaptive signatures by applying Population Branch Statistic (PBS) using the Han_Changchun and European from the HGDP as the ingroup and outgroup reference groups, which allowed identifying genes that were potentially under selection in GPH but not in Northern Han. One hundred fifty-five candidates were detected after filtering the top 0.001 percentile. Among them, one of the most vital PBS selection signals was located on chromosome 2, which comprises eleven genes [*HADHA*, *LRPPRC*, *ectodysplasin A receptor* (*EDAR*), and *ITGA6*, etc.]. Hydroxyacyl-CoA dehydrogenase trifunctional multienzyme complex subunit alpha (*HADHA*), also known as tri-functional protein alpha, a monolysocardiolipin acyltransferase-like enzyme, is essential for fatty acid beta-oxidation and cardiolipin remodeling and plays a vital role in functional mitochondria in heart organism of human beings [55]. Leucine-rich pentatricopeptide repeat containing (*LRPPRC*) is a mitochondria protein that maintains the stability of the mitochondrial transcriptome [56]. We also observed the *EDAR*, the identified biological targets influenced by mutation, associated with multiple phenotypes, including the shovel shape of upper incisors, mammary and eccrine glands, and hair straightness [57–59]. Previous studies also found that *EDAR* is related to facial characteristics in Uyghurs [60]. To further elucidate the population-specific biological adaptation for ethnolinguistic groups in Guangxi Province, we changed the selected groups of the trios mentioned above as (Zhuang/Miao/Jing/Sui/Yao_Guangxi)-Han_Changchun-European and the top 0.001 percentile distribution of PBS were compared with GPH (Fig. 7a). See Additional file 2: Table S9 for more details. The number of selected genes for Zhuang, Miao, Yao, Sui, and Jing were 143, 153, 144, 153, and 169, respectively. We also analyzed genes shared between populations subject to natural selection. For example, one of the significant PBS values was interleukin-6 (*IL6*), which encodes interleukin-6 and is the indicator of malaria severity, which was selected in the Sui, Jing, Miao, and Zhuang groups (Additional file 2: Table S9). The HLA system, which plays a significance role in the outcome of infectious diseases including malaria parasite, was also identified based on the XPEHH method using Guangxi ethnic minorities (Zhuang/Yao/Sui/Jing) and Mongolian as target and reference groups, respectively (Additional file 2: Table S10). Notably, *FADS2* was identified in Zhuang, Yao, Sui, and Jing populations but not in GPH. This gene produces the fatty acid desaturase (*FADS*) enzymes that facilitate the synthesis of medium-chain (MC)-PUFA precursors into long-chain polyunsaturated fatty acids (LC-PUFAs). Many SNPs were included in the *FADS* region, suggesting they were target for strong selection. Among these, rs174570 is connected to the levels of total cholesterol (TC), low-density lipoprotein (LDL), and high-density lipoprotein (HDL) in Europeans’ blood [61]. Analysis of six SNPs, including rs174570 in the Greenlanders, revealed that these loci were marginally related to multiple phenotypes [62]. We found that rs174570 within FADS2 showed a strong selection signature in our study (Fig. 7b). Meanwhile, an ancestral haplotype (the C allele of rs174547) was fixed at a high frequency in the Flores sample in line with a recent selective sweep. High frequencies of the ancestral allele are present in other Southeast Asian groups as well, indicating strong selection in their common ancestor followed by drift and further selection occurring in island Southeast Asian populations [63]. Here, the ancestral allele was observed at a high frequency in GPH as well as in other East Asian groups, especially among the Hainan Li populations, with the highest frequency. And there was a decreasing trend from south to north for the ancestral allele of rs174547 (Fig. 7d). The same pattern was also observed in the other SNP loci, including two loci for *FADS2* variants (rs174570 C/T and rs1535 G/A) (Fig. 7c and e). We further explored the allele frequency distribution of *FADS*-related mutations in spatiotemporally diverse Chinese people and found that ASEA harbored a high proportion of ancestral alleles, suggesting ancient people in South China had a diet rich in animal protein (Additional file 2: Table S11).

We also calculated PBS for GPH using ethnolinguistically different Guangxi groups and Northern Han as the second and third populations to characterize differentiated regional-specific adaptation. Many alleles exhibited strong regional-specific structures with GPH as the target group when we restricted the condition to the top 0.001 percentile but were less explored in the genetic study (Additional file 1: Fig. S18a; Additional file 2: Table S12). We observed complex fine-scale population structures and different natural adaptation signatures among six groups in Guangxi, which promoted discrepancies in allele frequencies among populations. We, therefore, explored highly differentiated variants (HDVs) with significantly different (top 0.001 percentile) allele frequencies between GPH and five ethnic groups in Guangxi Province (Additional file 1: Fig. S18b). We annotated these identified HDVs using the ClinVar Database [65] and the Variant Effect Prediction (VEP) tool [64]. We highlighted the five selected loci annotated as pathogenic or likely pathogenic (levels 5 and 4, respectively) in the ClinVar Database to map the frequency distribution of the six Guangxi populations (Fig. 7f). These five pathogenetic HDV loci are associated with the susceptibility of osteopetrosis (*CLCN7*), hearing loss (*GJB2*), cardiomyopathy (*PRDM16*), arrhythmogenic right ventricular dysplasia 1 (*TGFB3*), and sucrase-isomaltase deficiency (*SI*), respectively. We observed significant differences in the frequency distribution of the six Guangxi populations at these five loci (the frequency of these loci in Miao_Guangxi was zero), indicating remarkable differences in medical-relevant variants among different groups in the same region. Findings from the medical relevance highlighted that specific clinical strategies should be implemented in the precision prediction and interpretation period.

## Discussion

### Different demographic history and complex population structure within ethno-linguistically diverse Guangxi groups

South China harbored complex societies based on rice farming as one of the agricultural origin centers in China. Until now, their genetic histories have mainly been uncharacterized, and their genetic origin and precise relationship to ancient humans and other modern East Asian groups have yet to be fully addressed. The lack of resolution may be due to the limited density of the genomic dataset and sampling. This study analyzed genome-wide SNP data from 619 present-day individuals, 261 of which were newly generated. We unraveled the population structure of GPH populations. We found that they harbored close relationships with Southern Chinese groups (including TK, HM, and Southern Han populations) from the PCA, Fst, outgroup-*f*_*3*_ statistics, and IBD analysis results. He et al. focused on the Tujia and Central Han Chinese, which have revealed strong genetic assimilation between Tujia people and Central Han Chinese [42], suggesting frequent genetic interactions among geographically close populations. Genetic homogeneity within GPH was also confirmed via the observed result from *f*_4_ (GPH1, GPH2; reference populations, Mbuti) and pairwise coincidence matrix, although the distinctions between Northern and Southern Han.

A previous study on mitochondria and the Y-chromosomes concluded that GPH did not descend from Han Chinese. It merely assimilated the Han Chinese language and culture [36]. In our study, we observed that GPH exhibited primary admixture with genetic components resembling AN-related and TB-related groups (Fig. 1b) and the Northern Han-related component varied within geographically differentiated GPH without mirroring geography (Fig. 2). This composition aligns with the north-south genetic gradients previously seen in Han Chinese populations and is consistent with the three major historical events of the north-south migration [10]. Furthermore, we explored the most potential genetic ancestry and reconstructed the fitted-best model of GPH by conducting admixture *f*_*3*_-/*f*_*4*_ statistics analysis, qpGraph, and qpAdm. The result indicated that GPH could be modeled as a mixed descendant of Northern Han and Southern TK indigenous communities (Fig. 3c) or simulated as a mixture of Northern millet farmers in the YRB and ancient groups from Taiwan (Fig. 3d). The distribution of haplotypes also supported north-south admixture (Additional file 1: Fig. S15). The mixed north-south pattern was also observed in other Southern Hans. For example, He et al. have analyzed the Southernmost Han Chinese on Hainan Island and inferred that the TK groups contributed about half of the ancestry into Hainan Han populations, with another ancestry related to Northern Han [41]. To clarify the proportion and date of the admixture that occurred in GPH, we conducted qpAdm, taken together with the result, the genetic profile of GPH is well simulated by two-way admixture models with ANEA/NEA and groups with ASEA/SEA ancestry (Fig. 4b, c). The distinct admixture pattern observed between the distal and proximal model could be explained by more gene flow from Northern Han into modern Southern ethnic minorities than that of ASEA. Besides, the unique ancestral component proportions of SEA we observed were different from those of other Han Chinese. We supposed that it could be the Han Chinese in the North who, after experiencing the southward migration and frequent interactions with Southern indigenous populations, formed the current GPH group. ALDER’s identified date of admixture was around 985.38 BCE–458.82 CE (Additional file 2: Table S8). Notably, this time coincided with the Qin Dynasty’s unification (206 BCE), which facilitated the migration and interactions between Northern and Southern groups in China. Subsequently, intergroup interaction was further promoted by three major southward migrations of Han Chinese populations (the Yongjia Rebellion, An-shi Rebellion, and Jingkang Rebellion). Besides, the ancestors who spoke AN in Southeast Asia received gene flow from China around 2500 years ago, consistent with Southern China’s and Northern Vietnam’s unification during the Qin and Western Han dynasties [66]. However, Fu et al. have clarified that admixture involving ANEA ancestry (ancient Northern East Asians) spreading across Southern East Asia occurred after the Neolithic. The spread of ANEA ancestry also increased genetic interactions in both directions, suggesting continuous genetic interactions between different cultural communities. Nevertheless, the long interval between historical and present events, intermediate demographic events, and statistical uncertainty could bias the interpreting of present-day genetic data in a historical context. To end this, the precise genetic relationship and date of admixture among different groups need further broader analysis covering larger amounts of ancient genomes to better verify the results observed with current datasets. Besides, we should pay more attention to the fact that the dates estimated by fastGLOBETROTTER and ALDER are at odds with each other. This was caused by the different theories applied to fastGLOBETROTTER and ALDER. The fastGLOBETROTTER is an efficient haplotype-based technique to date admixture events, while the ALDER is an approach that exploits the exponential decay of admixture-induced linkage disequilibrium to infer admixture histories [67, 68]. The other statistical technique innovations with high-deep sequencing data should be developed to validate and revise our findings. The fine-scale genetic structure within East Asian populations suggested that GPH was more closely related to Guangxi indigenous groups compared to other Han populations (Fig. 5g). But fine-scale analyses focused only on 619 individuals covering five indigenous communities and one sampled population in Guangxi revealed the complex demographic history and striking differences between GPH and other Southern indigenous groups, which did not support that GPH was a Southern indigenous group but rather an admixture of Northern Han who migrated southwards and local indigenous people (Fig. 6a–g).

### Local adaptation of linguistically diverse Southern Chinese populations

Here, we have attempted to understand the genetic basis of local adaptation to Guangxi populations. We first searched for the population-specific signature of selection and identified several genes with extreme significance, including *HADHA*, *LRPPRC*, *EDAR*, etc. *HADHA* is a monolysocardiolipin acyltransferase-like enzyme associated with the functional mitochondria in human heart tissues. *LRPPRC*, a known gene that causes Leigh syndrome [69], is a mitochondrial protein that binds with its protein partner *SLIRP* and keeps the transcriptome of the mitochondria steady [56]. Previous studies have revealed the pleiotropic nature of *EDAR*, including the shovel shape of upper incisors, hair straightness, facial characteristic mammary, and eccrine gland [57–60]. For example, shovel-shaped upper incisors were inferred to be widely spread across Native American groups. However, they were most common in Asian populations compared to Africans and Europeans, which had a geographic distribution analogous to that of a derived variant 1540C (rs3827760). This allele is strongly associated with the tooth-shoveling grade as the number increases, explaining one fourth of this phenotype’s heritability and suggesting that multiple elements are involved in dental traits [59]. Population-specific natural adaptation signals associated with immunity, malaria resistance, etc., were identified in other Guangxi minorities, which is in line with those previously observed in Hainan Li populations [70]. Interestingly, *FADS* (*FADS1* and *FADS2*) were observed when we conducted a population-specific analysis of Guangxi ethnic minorities (Zhuang/Yao/Sui/Jing). *FADS1* and *FADS2* encode delta-5 and delta-6, respectively, which limit the transformation from linoleic acid (omega-6) and α-linolenic acid (omega-3) to eicosapentaenoic acid (EPA and omega-3), docosahexaenoic acid (DHA and omega-3), and arachidonic acid (omega-6). Previous studies have sought to explore the genetic basis of human adaptation to different diets, especially high-lipid diets, and *FADS* is the gene most related to lipid-rich diet environment found in Greenlandic Inuit and Flores groups [62, 63]. Multiple SNPs have been identified in the *FADS* region. For example, rs174570 is related to TC, LDL, HDL, and triglyceride levels in Europeans’ blood [61]. Another SNP, rs174547, is close to fixed in Flores samples in high frequency with the ancestral allele C. The same pattern of high-frequency distribution of ancestral alleles was also observed in other Southeast Asian populations as a result of common ancestry. Here, we also observed the similar allele frequency distribution of the ancestral allele C in GPH and other East Asian groups. Therefore, we speculated that this might be attributed to positive selection that occurred in their common ancestors, followed by multiple admixtures with each other and additional selection to various local environments. We also observed one SNP (rs174547 C/T) allele frequency distribution of *FADS1* and two SNPs (rs174570 C/T and rs1535 G/A) of *FADS2* showed a gradual decrease from south to north, which may be associated with natural selection or the northward migration of the ancestral populations (Fig. 7c–e). Significant differences in the dietary structure of coastal and inland groups can also have influenced the distribution of frequencies. Besides, HDVs were also identified in our work as being related to hearing loss, skeletal development, and other complex diseases or traits (Fig. 7f). Our finding showed that implementing WGS-level data in ethnolinguistically different groups will promote precision medicine in larger-scale populations with different genetic backgrounds.

## Conclusions

This study has characterized fine-scale population structure, complex demographic history, and differentiated local adaptations of GPH and their ethnolinguistically diverse neighbors. We found that GPH was genetically related to neighboring Guangxi TK, HM, and Haikou Han groups, suggesting extensive admixture among Han and indigenous populations in South China. Our estimated admixture time and constructed admixture model of GPH provided evidence for the admixture hypothesis of GPH, suggesting that GPH can be modeled as an admixture of ancient Northern group which associated with YRB farmers and a Southern group related to the ancient people of Taiwan. Furthermore, we revealed extensive differences in genetic diversity between GPH and other Southern aboriginals and highlighted language-related population substructure in South China. Finally, we have identified many population-specific and regional-specific natural selection signals associated with the living circumstances and pathogen exposure in Guangxi people, including *FADS* associated with lipid-rich diet practice and other genes related to immunity and malaria resistance. We also observed medically relevant variations related to hearing loss and other complex clinical diseases or traits.

## Methods

### Sample collections, DNA preparation, and genotyping

A total of 619 unrelated individuals from 56 populations were included here, 358 of which were previously genotyped with Affymetrix Array [39, 40, 43, 71]. Here, we newly collected saliva samples from 236 unrelated healthy Han individuals from Guangxi Province living in Baise (3), Guangnan (3), Wanwei (3), Laibin (13), Chongzuo (11), Fangchenggang (5), Jingxi (5), Tianlin (5), Tianyang (5), Guigang (24), Hechi (20), Qinzhou (14), Hezhou (15), Beihai (16), Liuzhou (17), Nanning (18), Wuzhou (18), Yulin (19), and Guilin (22), and 25 unrelated Han individuals recruited from Yunnan Province living in Wenshan Zhuang and Miao Autonomous Prefecture. We obtained all samples with written informed consent and afforded genetic testing results based on their ancestral makeup and genetic health condition. The geographic distribution of sampling locations is shown (Additional file 1: Fig. S1). All included individuals were required to be indigenous residents with at least three generations of history in the sampling sites and the offspring of a non-consanguineous marriage between the geographically close Miao and Zhuang populations or any other ethnic minorities. Our project and corresponding protocols were reviewed and approved by the Medical Ethics Committees of West China Hospital of Sichuan University (2023-306) and the Ministry of Science and Technology of the Human Genetic Resources Administration of China (HGRAC) with the registration number 2024BAT00173. All procedures were performed following the regulations of the HGRAC and ethical principles recommended by the Declaration of Helsinki [72]. The extraction and purification of human genomic DNA (gDNA) were performed using the QIAamp DNA Mini Kit (QIAGEN, Germany), and then the quantification was conducted using the Qubit dsDNA HS Assay Kit (Thermo Fisher Scientific) on an Invitrogen Qubit 3.0 fluorometer following the manufactures’ instructions. The gDNA was subsequently preserved at −20 °C until the amplification. All samples were genotyped using Affymetrix Array.

### Quality control and reference dataset assembly

The generated data we then used PLINK v.1.9 [48] to obtain the quality-controlled raw data before combined with publicly available data. Missing SNPs and individuals were identified using the parameters of --geno 0.05 and --mind 0.05. We used King to remove related samples within third generations [73], producing a final dataset including 465,941 SNPs for subsequent analyses. Public data from the HGDP [74] and Oceania genomic resource [12], AADR (HO dataset and 1240 K dataset) [75] from David Reich Lab [76], and our previously reported populations genotyped via Affymetrix Array chip [26, 39–42] were included to form the following four merged datasets. The high-density SNP dataset included population data from Miao, Jing, Zhuang, Li, Tujia, and Mongolian, which included 465,941 SNPs [40–43, 77, 78]. High-density global dataset included populations from HGDP and Oceania genomic resources, which included 424,501 SNPs. The high-density datasets were used to explore the complex genetic relationships or models based on the phased haplotype. We also merged our Affymetrix dataset with AADR datasets and formed the middle-density 1240 K (359,009 SNPs) and low-density HO (119,114 SNPs) datasets. The former was used to explore the genetic relationship between our focused populations and ancient Eurasians. The latter was used to explore the basic genetic patterns among all modern and ancient populations in the descriptive analysis.

### Data analysis

#### Inference of genetic relationship and Global ancestry

Smartpca package implemented in the EIGENSOFT v6.0.1 [79] was used to perform PCA analyses with the following parameters: numoutlieriter: 0 and lsqproject: YES. All included ancient populations from Russia [80, 81], Mongolia [10], Japan [10], and China [8, 10, 46] were projected onto the two-dimensional plots focused on their genetic variations of modern groups. We also performed PCA focused only on 619 individuals comprising six ethnic groups in Guangxi. We applied PLINK v.1.9 [48] to remove the strongly linked SNPs with these parameters: --indep-pairwise 200 25 0.4. We used a model-based clustering analysis with unsupervised mode and predefined ancestral populations between 2 and 20 to dissect the ancestral component of GPH and modern and ancient reference populations using ADMIXTURE 1.3.0 [82]. After calculating the cross-validation errors, we chose seven and twelve predefined ancestral sources in our study. We also performed ADMIXTURE for 619 individuals spanning six different groups.

#### Genetic distances and relationship analyses

PLINK v.1.9 [48, 83] and our in-house scripts were used to calculate the pairwise fixation indexes (Fst) [84], which can be used to evaluate the genetic differences or similarities between our studied populations and other Eastern Asian populations. Our study also constructed a phylogenetic tree based on the results of pairwise Fst. We conducted various types of *f*_3_ and *f*_4_ statistics using the qp3Pop and qpDstat programs built-in ADMIXTOOLS v7.0.2 [85] with default settings. Outgroup-*f*_3_ statistics in the form of *f*_*3*_ (X, Y; outgroup) was used to assess the genetic affinity between X and Y, where X represented GPH populations and Y represented the different ancient and modern East Asian populations. The larger estimated *f*_*3*_ value between the two populations indicated the more shared genetic drift. Next, we performed admixture-*f*_3_ statistics in the form of *f*_*3*_ (Source1, Source2; GPH) to explore the potential admixture ancestry candidates, which contributed to different degree of gene flows in studied populations. Finally, *f*_4_ statistics in the form of *f*_*4*_ (reference1, reference2; GPH, outgroup), *f*_4_ (reference1, GPH; reference2, outgroup), and *f*_*4*_ (GPH1, GPH2; reference, outgroup) were conducted to evaluate the differentiated genetic affinities between target and reference populations, the comparison of differences between groups and the confirmation of possible ancestral groups, and the homogeneity within targets respectively.

#### Admixture graph reconstruction via TreeMix plus qpGraph

We constructed the phylogenetic tree based on the allele frequency using unsupervised clustering analysis of TreeMix v.1.13 [86] with the default parameters to generate the best-fitted model with migration events ranging from 0 to 3 and explore the phylogenetic relationships among 29 modern East Asian populations. To further explore one successful admixture model that fits genomic data best, we carried out qpGraph analysis [85, 87] with the following settings: diag: 0.0001; lsqmode: NO; hires: YES; blgsize: 0.05; precision: 0.0001; initmix: 1,000; zthresh: 0; useallsnps: NO; terse: NO. Moreover, we successfully constructed the genomic history of our target populations based on the basic model [88].

#### Analyses of mixture proportions via qpWave/qpAdm

We estimated the minimum number of ancestry sources of our studied populations and assessed the genetic heterozygosity between GPH and ancestral sources using qpWave and rank tests and calculated corresponding admixture proportions using qpAdm [89] packages in the ADMIXTOOLS. We conducted qpAdm modeling in two different stages. In the first stage, we searched for the populations, which included groups from the Neolithic to the Iron Age. We used seven populations as outgroup populations (Mbuti, UstIshim, Kostenki14, GoyetQ1161_N, Villabruna, Natufian and Mixe) which were used by Vikas Kumar et al. [49]. For proximal sources, we looked for the source populations that are close temporally to the studied populations from Guangxi, and we selected nine worldwide populations (Mbuti, Kostenki14, Ust_Ishim, Onge, Australian, MA1, Mixe, Atayal, and Papuan) as the outgroup populations. We used one additional outgroups population (Yakut) in the three-way admixture model. One additional parameter, “allsnps: YES”, was used here. The best-fitting qpAdm model has followed these principles: (1) negative ancestry proportion did not exist, (2) the *p* value of the rank test must be more than 0.05, and (3) the standard error was smaller than the minimum mixture proportion.

#### Recent demographic history inference and IBD analyses

We performed haplotype phasing using a statistical-based method built in the SHAPEIT v2.r90 [90] before we inferred recent demographic histories using IBDNe v23Apr20 [91]. IBDNe was used to estimate the effective population size from 1 to 150 generations ago. Based on the phased dataset, we calculated the pairwise shared IBD within and between populations using Refined IBD with the short gaps removed [47]. We calculated different IBD lengths shared between groups, including less 1 cM, 1–5 cM, 5–10 cM, over 10 cM, and the total length, where cases less than 1 cM were considered as noise signals and were not included in the analysis.

#### Genetic diversity estimation

We estimated ROH to measure the consanguinity among the populations in the merged HO dataset using PLINK v.1.90 [48] after removing single-nucleotide variants (SNVs) with minor allele frequency (MAF) < 0.05 or missing rate lager than 0.05 using a window of 500 kb to scan for ROHs throughout the genome and allowing one heterozygous for per window. To further explore the information included in ROH, we classified ROH into three different classes: short (less than 1 Mb), medium (1–5 Mb), and long ROH (larger than 5 Mb).

#### Dating admixture events and fineSTRUCTURE analysis

ALDER [67] was used for estimating the dates scale of population admixture events using the two parameters: mindis: 0.005, jackknife: YES. We also painted the recipient chromosomes for our target individuals using a statistical algorithm implemented in the ChromoPainter v2 and ChromoCombine v2 [51] with all other available donor chromosomes served as the potential donors and conducted the fine-scale genetic structure based on the result of ChromoPainter using the fineSTRUCTURE v4 [92]. We used fastGLOBETROTTER (34) based on the shared haplotype information and used 14 genetically distinct populations (Sui_Guangxi, Miao_Guangxi, Han_Harbin, Han_Shenyang, Hlai_Qiongzhong, Cambodian, Dai, Daur, Lahu, Oroqen, Yakut, Basque, French and Pathan) as surrogates following the default setting to further identify ancestry sources and the date and characterize the admixture events.

#### Natural selection signal identification

Population Branch Statistic (PBS) was used to detect population-specific natural selection signals in GPH and different Guangxi ethnic minorities. We used Han_Changchun as an ingroup and European as an outgroup to estimate the ancient selection signatures in Southern Chinese populations. Notably, we identified Zhuang, Yao, Sui, and Jing-specific signatures using this strategy. To validate the various adaptive genes specific to Guangxi ethnic minorities inferred by the PBS approach, we also estimated cross-population extended haplotype homozygosity (XPEHH) of significant genes under adaptive evolution (*p* < 0.01) for Guangxi ethnic minorities using selscan v1.2.0 [93], in which we used ethnolinguistically diverse Chinese papulations as the reference population. We also explored the regional-specific adaptive signals using GPH as the target population and GXZ, Yao, Sui, Jing, and Northern Han populations from China as the second and third references, respectively. We calculated the allele frequency of selected alleles using the 10K Chinese People Genomic Diversity Project (10K_CPGDP).

#### Y-chromosomal and mtDNA haplogroup analysis

Y-chromosomal and mitochondrial SNPs (Y-SNP) were extracted from the high-density dataset to explore the paternal and maternal population history. We conducted quality control on the merged dataset using PLINK v1.9 (2 Apr 2022) with two parameters (--geno: 0.1 and --mind: 0.1) [48]. After filtering, we retained 5162 Y-SNPs and 1124 mitochondrial DNA (mtDNA) SNPs for uniparental demographic history construction. Then, Y-chromosomal haplogroup was classified using the python package of hGrpr2.py implemented in HaploGrouper [94] and LineageTracker [95] with two additional files used in the HaploGrouper-based analysis (treeFileNEW_isogg2019.txt and snpFile_b38_isogg2019.txt). For maternal haplogroup classification, HaploGrouper and Haplogrep were used [94]. We used BEAST2.0 [96] and LineageTracker [95] to rebuild the paternal phylogeny and exhibit the population split, expansion, and admixture events. Three additional software (EAUti, Tracer v1.7.2 and FigTree v1.4.4) were used to generate the input files for BEAST-based analysis and visualize the phylogeny tree. We also used BEAST2.0 to reconstruct the maternal phylogeny topology. Eventually, median-joining network built in the popART [97] was used to reconstruct the network relationship based on the genetic variations obtained from maternal and paternal analyses.

#### Highly differentiated variant discovery and annotation

We used PLINK v.1.9 [48] to identify highly differentiated locus, and variant annotation was conducted using Ensembl Variant Effect Predictor (VEP) [98] and clinical importance from the ClinVar database [65].

### Supplementary Information

**Additional file 1: Fig. S1.** Sample location of the GPH. **Fig. S2.** Genetic affinities of the GPHs based on the pairwise Fst and shared genetic drift. **Fig. S3.** Pairwise identity-by-descent (IBD) networks within the East Asian groups. **Fig. S4.** Genetic affinities between GPH and 30 ancient Chinese populations. **Fig. S5.** Affinity testing of ancient populations. **Fig. S6.** F_4_-statistic test for gene introgression in GPH. **Fig. S7.** Asymmetric testing of GPHs. **Fig. S8.** Allele frequency-based asymmetrical *f*_4_ statistics test. **Fig. S9.** Runs of homozygosity (ROH) of the populations in East Asia. **Fig. S10.** Inferred phylogenetic relationship using TreeMix. **Fig. S11.** Overview of inferred admixture for GPH using fastGLOBETROTTER. **Fig. S12.** Effective population sizes of Guangxi groups inferred from IBDNe. **Fig. S13.** Fine-scale genetic structure and coancestry curves based on the shared haplotype data. **Fig. S14.** The population structure of five ethnic communities and GPH in Guangxi Province. **Fig. S15.** Paternal lineages in the context of East Asia. **Fig. S16.** Y chromosome haplogroup distribution. **Fig. S17.** Mitochondrial haplogroup distribution. **Fig. S18.** Natural selection signals and highly differentiated loci.

**Additional file 2: Table S1.** Symmetric *f*_4_-statistic testing. **Table S2.** Pairwise qpWave results indicated the genetic heterozygosity between GPH and East Asians. **Table S3.** Identification of modern Northern ancestral sources in the form of *f*_4_(reference populations, GPH; Northern ancestral sources, Mbuti). **Table S4.** Modern ancestral uniqueness of GPH determined by *f*_4_-statistic in the form *f*_4_(Northern ancestral sources, GPH; reference populations, Mbuti). **Table S5.** Pairwise qpWave results confirmed the genetic heterozygosity between GPH and ancestral sources in the distal qpAdm model. **Table S6.** Comparing genetic homogeneity between GPH and ancestral surrogate populations used in proximal qpAdm model using qpWave. **Table S7.** The three-population mixing model of GPH inferred by qpAdm. **Table S8.** Admixture times inferred from ALDER for GPH. **Table S9.** The population-specific adaptive signals using PBS in the form of (GPH/Miao/Yao/Zhuang/Sui/Jing_Guangxi-Han_Changchun-European). **Table S10.** The result of XPEHH of GXZ. **Table S11.** Frequency distribution of the three loci of FADS in ancient East Asians. **Table S12.** The regional-specific adaptive signals in the form of (GPH-Jing/Miao/Sui/Yao/Zhuang_Guangxi-Han_Changchun) using PBS.

## Data Availability

All data generated or analyzed during this study are included in this published article, its supplementary information files, and publicly available repositories. The genome-wide variation data were obtained from the public dataset of Allen Ancient DNA Resource (AADR) (https://reich.hms.harvard.edu/allen-ancient-dna-resource-aadr-downloadable-genotypes-present-day-and-ancient-dna-data). The results of the analyses have been submitted in the supplementary materials and also deposited into the OMIX database (https://ngdc.cncb.ac.cn/omix/) through an accession number of OMIX005448. The allele frequency data derived from human samples have been deposited in the Zenodo (https://zenodo.org/records/10681844). The access and use of the data shall comply with the regulations of the People’s Republic of China on the administration of human genetic resources. Requests for access to data can be directed to Guanglin He (Guanglinhescu@163.com).

## Abbreviations

GPH: Guangxi Pinghua Han people
TK: Tai-Kadai
FADS: Fatty acid desaturases
GWAS: Genome-wide association studies
TOPMed: Trans-Omics for Precision Medicine
gnomAD: Genome Aggregation Database
WGS: Whole-genome sequencing
NIPT: Non-Invasive Prenatal Testing
ChinaMap: China Metabolic Analytics Project
WBBC: Westlake BioBank for Chinese
CKB: China Kadoorie Biobank
NSCLC: Chinese non-small cell lung cancer
TB: Tibeto-Burman
HM: Hmong-Mien
AN: Austronesian
AA: Austroasiatic
SNP: Single-nucleotide polymorphism
HGDP: Human Genome Diversity Project
HGDP-O: Human Genome Diversity Project and Oceania
HO: Human Origin
AADR: Allen Ancient DNA Resource
PCA: Principal component analysis
YRB: Yellow River Basin
ASEA: Ancient Southern East Asian
ANEA: Ancient Northern East Asian
GXZ: Guangxi Zhuang
NEA: Northern East Asian
SEA: Southern East Asian
IBD: Identity by descent
ROH: Runs of homozygosity
ALDER: Admixture-induced Linkage Disequilibrium for Evolutionary Relationship
NRY: Non-recombining Y-chromosome
PBS: Population Branch Statistic
HADHA: Hydroxyacyl-CoA dehydrogenase trifunctional multienzyme complex subunit alpha
LRPPRC: Leucine-rich pentatricopeptide repeat containing
EDAR: Ectodysplasin A receptor
IL6: Interleukin- 6
MC-PUFA: Medium-chain polyunsaturated fatty acids
LC-PUFAs: Long-chain polyunsaturated fatty acids
TC: Total cholesterol
LDL: Low-density lipoprotein
HDL: High-density lipoprotein
HDV: Highly differentiated variant
VEP: Variant effect prediction
SI: Sucrase-isomaltase
HGRAC: Human Genetic Resources Administration of China
gDNA: Genomic DNA
SNVs: Single-nucleotide variants
XPEHH: Cross-population extended haplotype homozygosity
Y-SNPs: Y-chromosomal SNPs
mtDNA: Mitochondrial DNA
