# Supplementary material for: Differentiated adaptative genetic architecture and language-related demographical history in South China inferred from 619 genomes from 56 populations
Source: BMC Biol. 2024 Mar 6;22:55. doi: 10.1186/s12915-024-01854-9 (PMC10918984; doi:10.1186/s12915-024-01854-9)
Supplement: Supplementary file 1 — Additional file 1: Fig. S1. Sample location of the GPH. Fig. S2. Genetic affinities of the GPHs based on the pairwise Fst and shared genetic drift. Fig. S3. Pairwise identity-by-descent (IBD) networks within the East Asian groups. Fig. S4. Genetic affinities between GPH and 30 ancient Chinese populations. Fig. S5. Affinity testing of ancient populations. Fig. S6. F4-statistic test for gene introgression in GPH. Fig. S7. Asymmetric testing of GPHs. Fig. S8. Allele frequency-based asymmetrical f4 statistics test. Fig. S9. Runs of homozygosity (ROH) of the populations in East Asia. Fig. S10. Inferred phylogenetic relationship using TreeMix. Fig. S11. Overview of inferred admixture for GPH using fastGLOBETROTTER. Fig. S12. Effective population sizes of Guangxi groups inferred from IBDNe. Fig. S13. Fine-scale genetic structure and coancestry curves based on the shared haplotype data. Fig. S14. The population structure of five ethnic communities and GPH in Guangxi Province. Fig. S15. Paternal lineages in the context of East Asia. Fig. S16. Y chromosome haplogroup distribution. Fig. S17. Mitochondrial haplogroup distribution. Fig. S18. Natural selection signals and highly differentiated loci. [file 12915_2024_1854_MOESM1_ESM.pdf]

Supplementary Figures

RESEARCH Open

Differentiated adaptative genetic architecture and language-related demographical history in South China inferred from 619 genomes from 56 populations

Qiuxia Sun[1,2,*], Mengge Wang[2,14,*#], Tao Lu[1,*], Shuhan Duan[3,2], Yan Liu[3,2], Jing Chen[2,5], Zhiyong Wang[2,6], Yuntao Sun[2,7], Xiangping Li[2,6], Shaomei Wang[2,8], Liuyi Lu[4,2], Liping Hu[6], Libing Yun[7], Junbao Yang[4], Jiangwei Yan[5], Shengjie Nie[6], Yanfeng Zhu[8], Gang Chen[12], Chuan-Chao Wang[13], Chao Liu[9,10,11], Guanglin He[2,14,*#] , Renkuan Tang[1,#]

Correspondence: Mengge Wang (Menggewang2021@163.com), Renkuan Tang (renktang2012@163.com), Guanglin He (guanglinhescu@163.com)

*Qiuxia Sun, Mengge Wang, Tao Lu and Guanglin He contributed equally to this work

[1]Department of Forensic Medicine, College of Basic Medicine, Chongqing Medical University, Chongqing, 400331, China
[2]Institute of Rare Diseases, West China Hospital of Sichuan University, Sichuan University, Chengdu, 610000, China
[3]School of Basic Medical Sciences, North Sichuan Medical College, Nanchong, 637100, China
[4]School of Clinical Medical Sciences, North Sichuan Medical College, Nanchong, 637100, China
[5]School of Forensic Medicine, Shanxi Medical University, Jinzhong, 030001, China
[6]School of Forensic Medicine, Kunming Medical University, Kunming, 650500, China
[7]West China School of Basic Science & Forensic Medicine, Sichuan University, Chengdu, 610041, China
[8]Department of Public Health, Chengdu Medical College, Chengdu, 610500, China
[9]Faculty of Forensic Medicine, Zhongshan School of Medicine, Sun Yat-sen University, Guangzhou, 510275, China
[10]Guangzhou Forensic Science Institute, Guangzhou, 510055, China
[11]Anti-Drug Technology Center of Guangdong Province, Guangzhou, 510230, China
[12]Hunan Key Lab of Bioinformatics, School of Computer Science and Engineering, Central South University, Changsha 410075, China
[13] State Key Laboratory of Cellular Stress Biology, National Institute for Data Science in Health and Medicine, School of Life Sciences, Xiamen University, Xiamen 361005, Fujian, China
[14]Center for Archaeological Science, Sichuan University, Chengdu, 610000, China

Contents

Figure. S1. Sample location of the GPH. 3

Figure. S2. Genetic affinities of the GPHs based on the pairwise Fst and shared genetic drift. 3

Figure. S3. Pairwise identity-by-descent (IBD) networks within the East Asian groups. 4

Figure. S4. Genetic affinities between GPH and 30 ancient Chinese populations. 4

Figure. S5. Affinity testing of ancient populations. 5

Figure. S6. F_4-statistic test for gene introgression in GPH. 6

Figure. S7. Asymmetric testing of GPHs. 7

Figure. S8. Allele frequency-based asymmetrical f_4-statistics test. 7

Figure. S9. Runs of homozygosity (ROH) of the populations in East Asia. 8

Figure. S10. Inferred phylogenetic relationship using TreeMix. 9

Figure. S11. Overview of inferred admixture for GPH using fastGLOBETROTTER. 9

Figure. S12. Effective population sizes of Guangxi groups inferred from IBDNe. 10

Figure. S13. Fine-scale genetic structure and coancestry curves based on the shared haplotype data. 11

Figure. S14. The population structure of five ethnic communities and GPH in Guangxi Province. 12

Figure. S15. Paternal lineages in the context of East Asia. 12

Figure. S16. Y chromosome haplogroup distribution. 13

Figure. S17.Mitochondrial haplogroup distribution. 14

Figure. S18. Natural selection signals and highly differentiated loci. 14

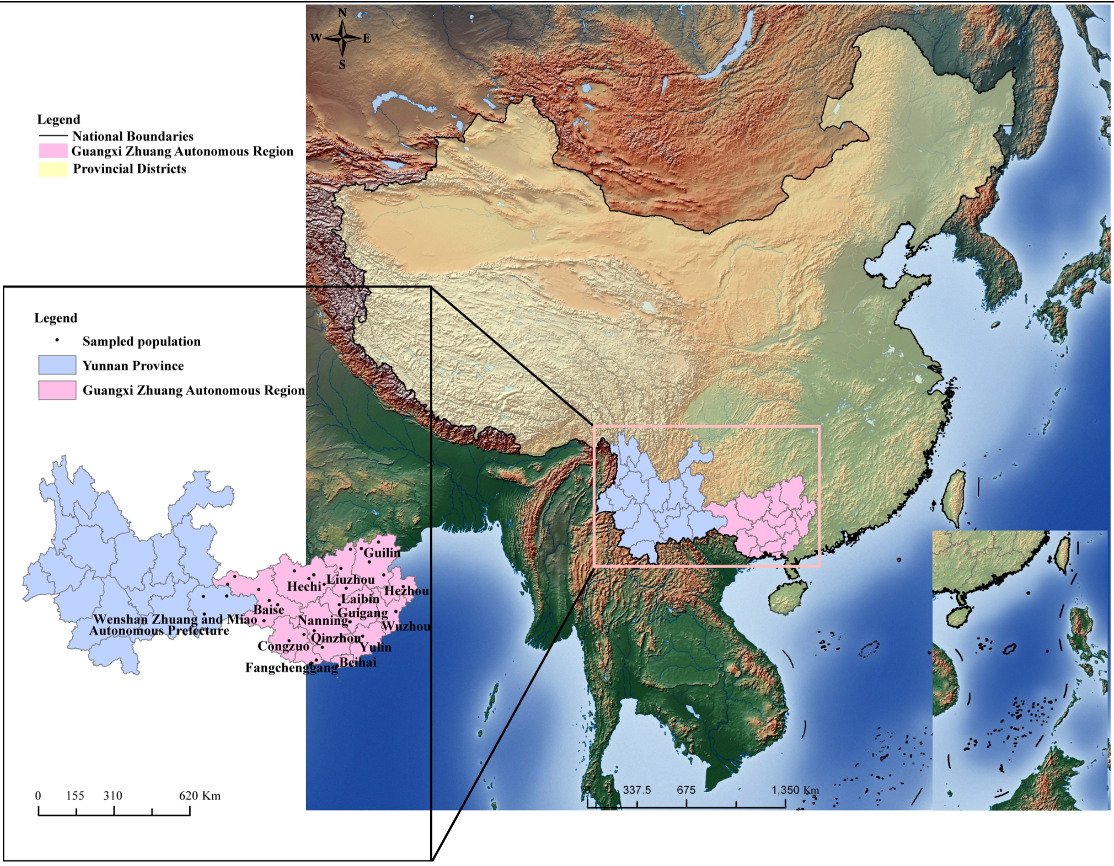

Figure. S1. Sample location of the GPH.

The geographic location of sampled GPHs in the current study includes 14 prefecture-level cities in Guangxi and one autonomous prefecture in Yunnan.

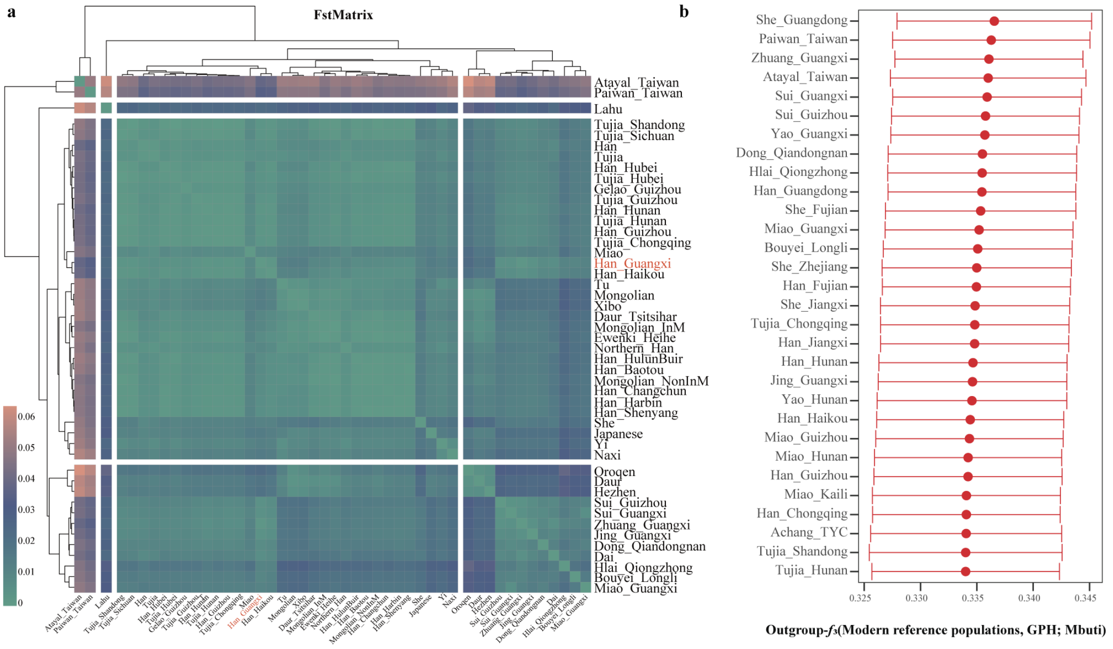

Figure. S2. Genetic affinities of the GPHs based on the pairwise Fst and shared genetic drift.

(a) Heatmap of pairwise Fst sharing among 47 East Asian populations in the merged HO dataset formed four genetic branches. The genetic distance between populations gradually increases from green to red. (b) Shared genetic drift with extreme significance (top 30) results for the outgroup-f_3-statistics in the form of f_3(modern reference populations, GPH; Mbuti).

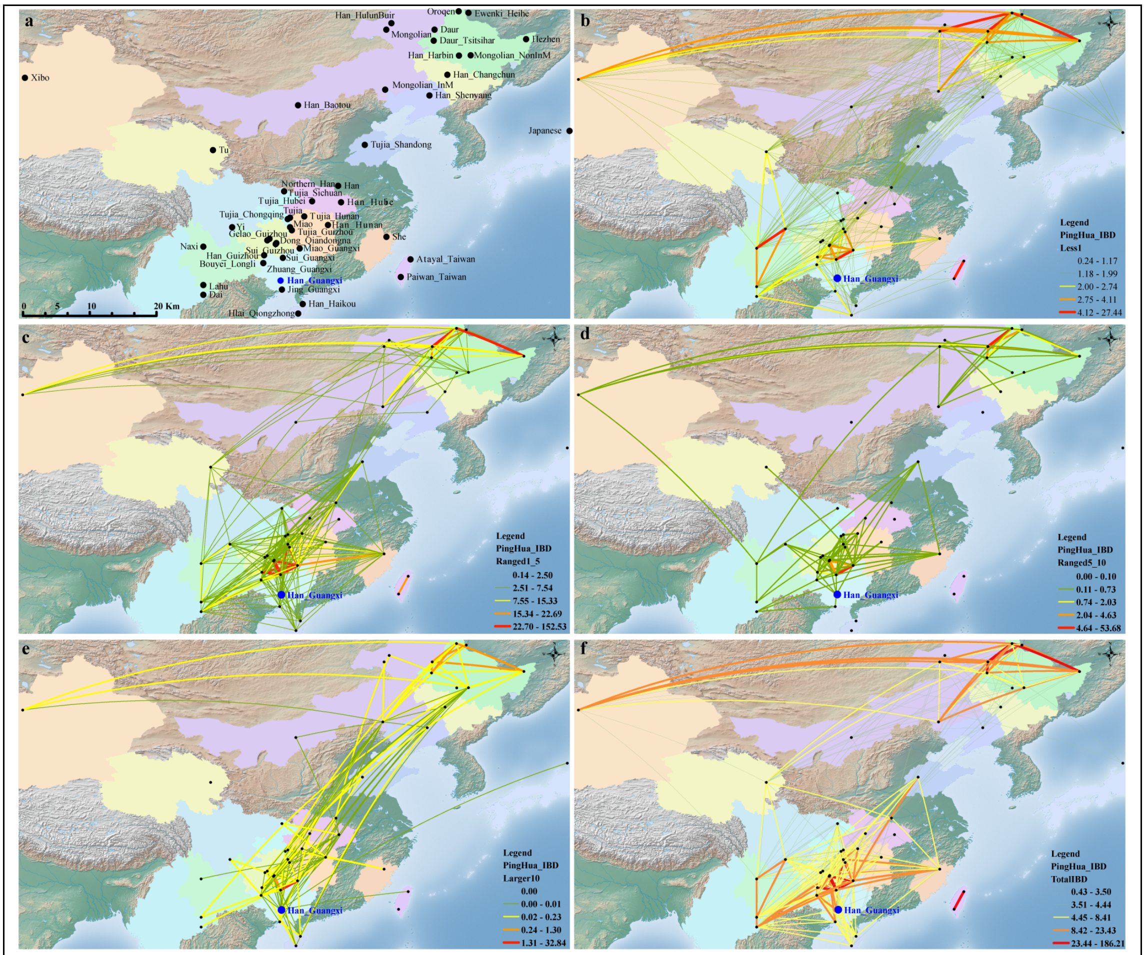

Figure. S3. Pairwise identity-by-descent (IBD) networks within the East Asian groups.
(a-f) IBD sharing models of population average IBD length in the range of less 1 cM, 1-5 cM, 5-10 cM, over 10 cM and total length among East Asian populations in the merged HO dataset. Different colors represent the different total number of shared IBD fragments between populations.

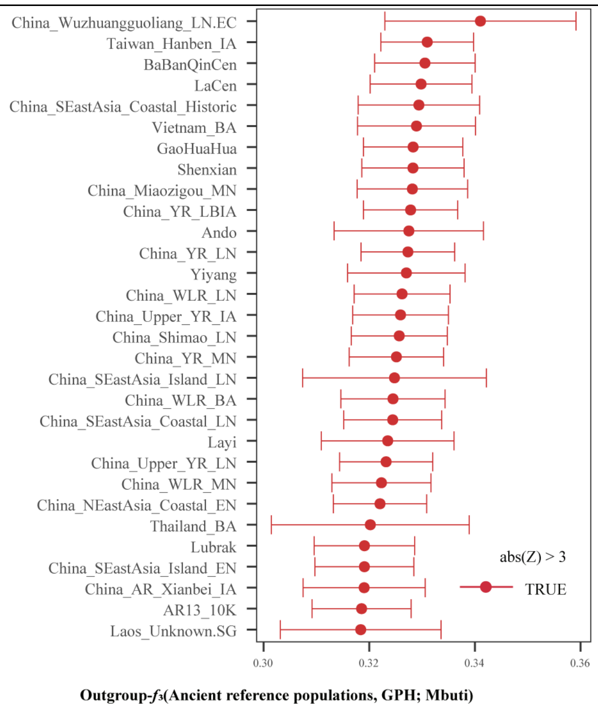

Figure. S4. Genetic affinities between GPH and 30 ancient Chinese populations.
Shared genetic drift with extreme significance (top 30) results for the outgroup-f_3-statistics in the form of f_3(ancient reference populations, GPH;

Mbuti).

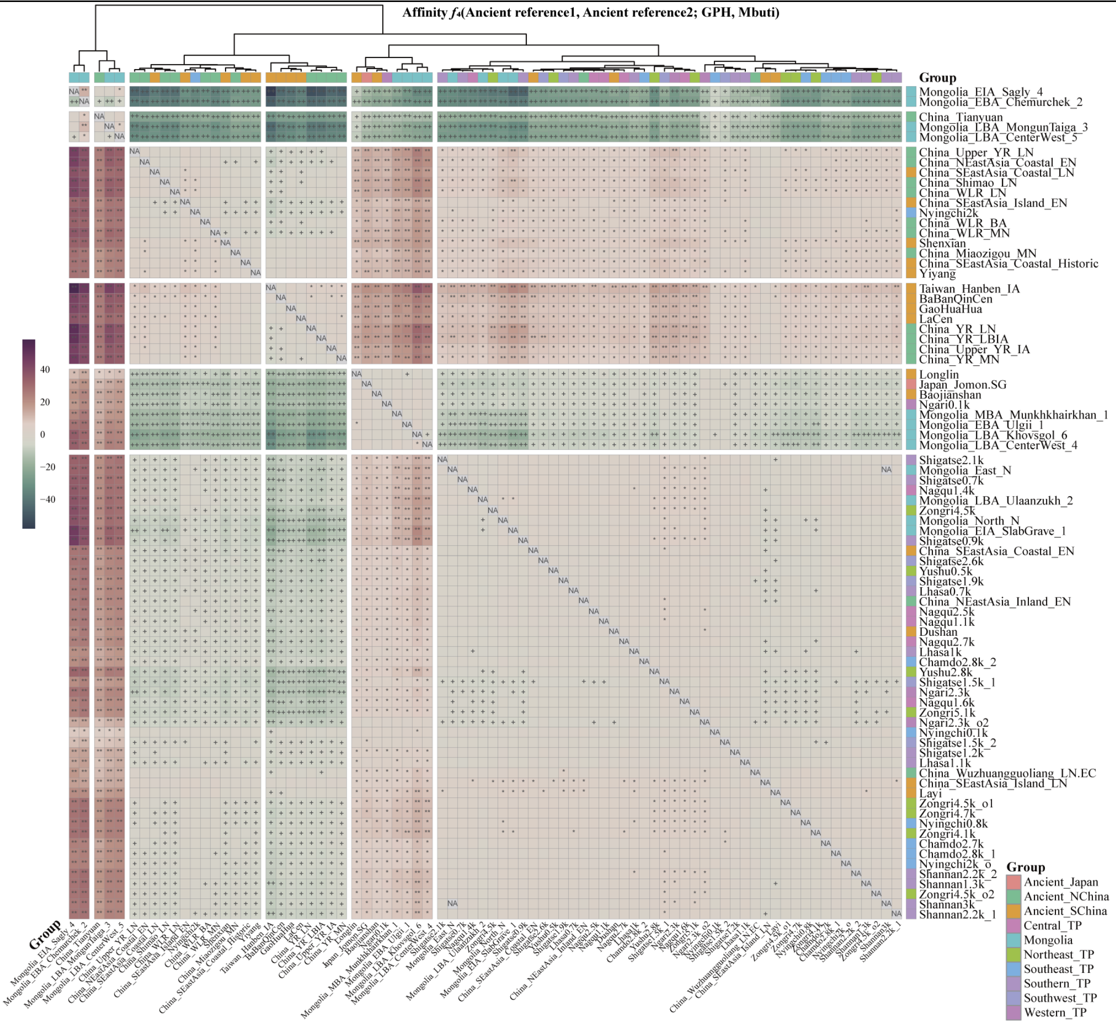

Figure. S5. Affinity testing of ancient populations.

The genetic affinity of GPH to different ancients was measured by the affinity f_4-statistic of f_4(ancient reference population1, ancient reference population2; GPH, Mbuti) (reddish brown and green indicate higher affinity).

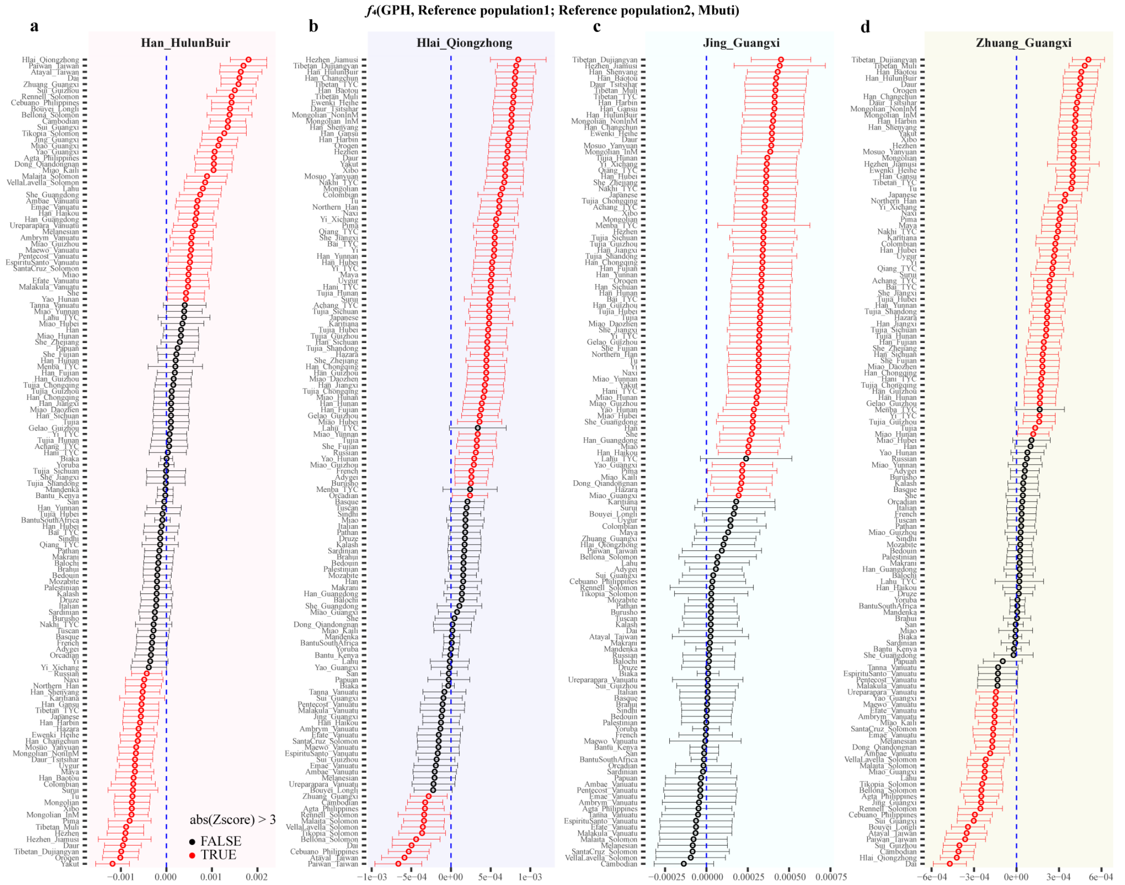

Figure. S6. F_4-statistic test for gene introgression in GPH.

(a-d) F_4-statistics in the form of f_4(GPH, reference population1; reference population2, Mbuti), where reference population1 represents the selected Northern Han (Han_HulunBuir) and Southern ethnic minorities (Hlai_Qiongzhong/Zhuang/Jing_Guangxi), reference population2 represents global groups in the merged HGDP data. Different pairwise population pairs were used to explore genetic differences between GPH and reference population1.

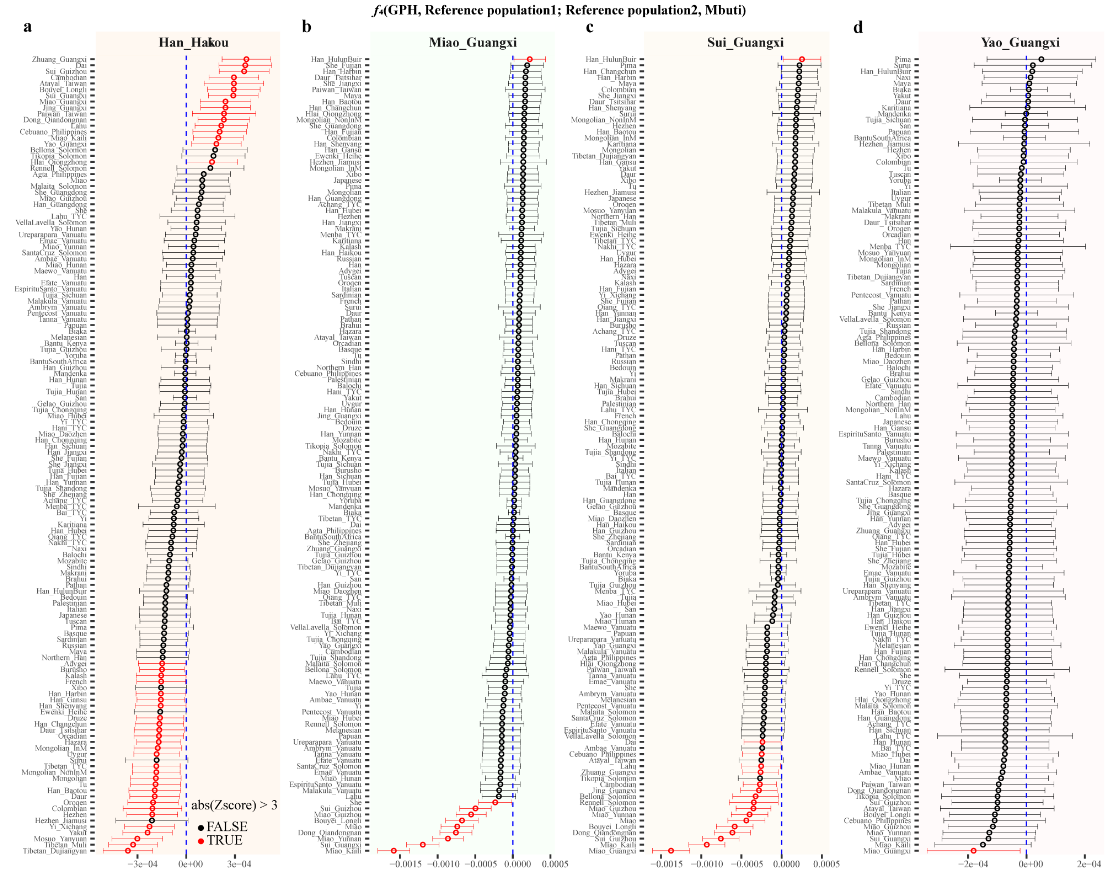

Figure. S7. Asymmetric testing of GPHs.

(a) The values of f_4(GPH, reference population1; reference population2, Mbuti) are shown with ±3 standard errors, where reference population1 represents Southern Han (Han_Haikou). GPH received more alleles from Southern ethnic minorities than Han_Haikou. (b-d) The asymmetric f_4-statistics of ethnic minority groups in Guangxi (Miao/Sui/Yao) as a target with different pairs of global reference populations. Significant negative values indicate that these groups affected more alleles from Southern ethnic minorities than those of GPH.

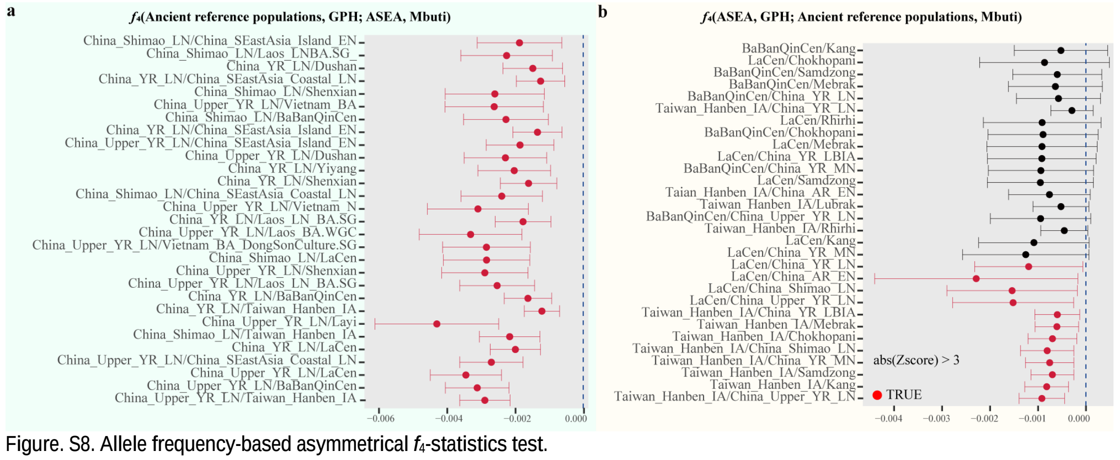

Figure. S8. Allele frequency-based asymmetrical f_4-statistics test.

(a) F$_4$-statistics in the form of f_4(ancient reference populations, GPH; ASEA, Mbuti) testing the GPH's genetic association with ancient Southern East Asian ancestries (ASEA) (including Taiwan_Hanben_IA, BaBanQinCen, LaCen, China_SEastAsia_Coastal_LN, Layi, Laos_LN_BA.SG, Shenxian, Vietnam_BA_DongSonCulture.SG, Laos_BA.WGC, Vietnam_N, Yiyang, Dushan, China_SEastAsia_Island_EN and Vietnam_BA) in contrast to other ancient East Asians. (b) Numbers of GPH ancestral sources were determined by f_4-statistics in the form of f_4(ASEA, GPH; ancient reference populations, Mbuti), where ASEA represents an identified ancestor (Taiwan_Hanben_IA, LaCen and BaBanQinCen) of GPH, ancient reference populations represent other ancient populations (the ancient people of East Asia, Southeast Asia and South Asia).

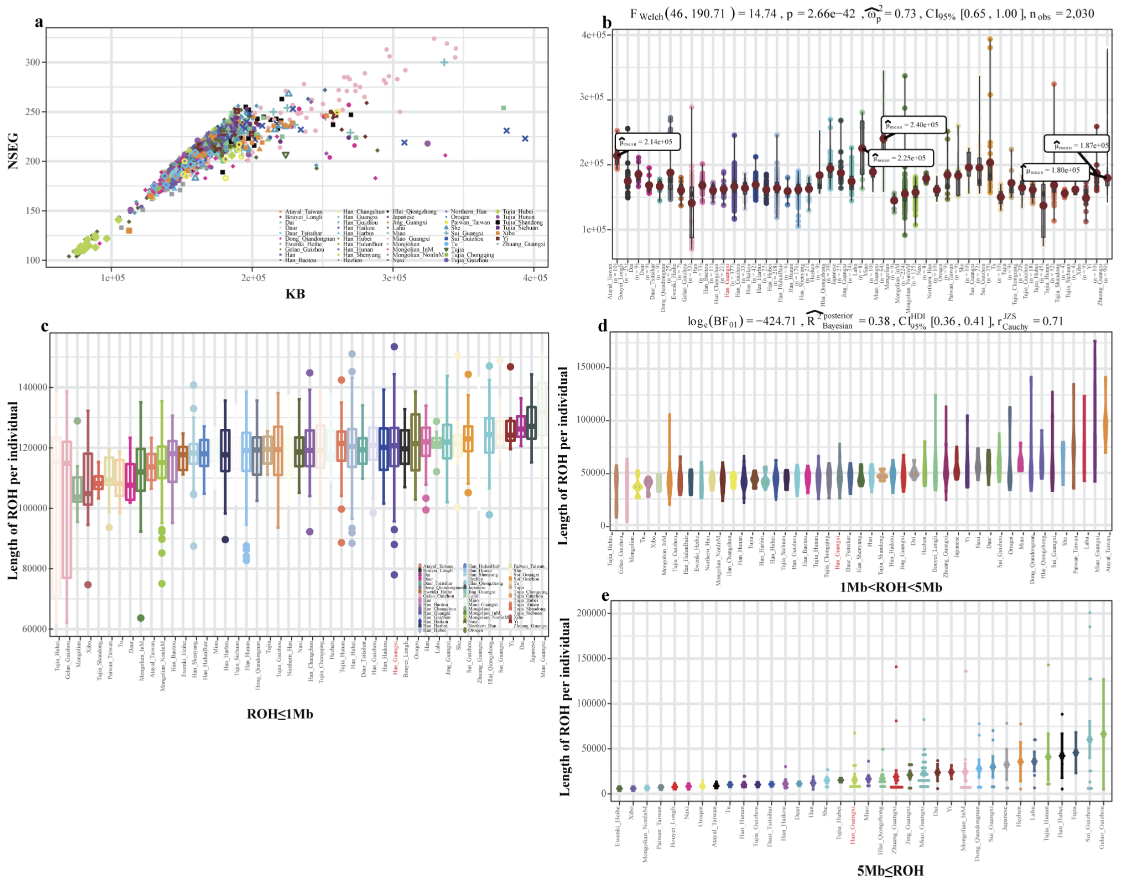

Figure. S9. Runs of homozygosity (ROH) of the populations in East Asia.

(a) Comparison of the total length of ROH (y-axis) and the total number of ROH (x-axis) in the population of the merged HO dataset. (b) The total length of ROH at the population level. (c-e) The total length of short (ROH ≤ 1Mb), medium (1Mb < ROH < 5Mb) and long (ROH≥5Mb) in different populations.

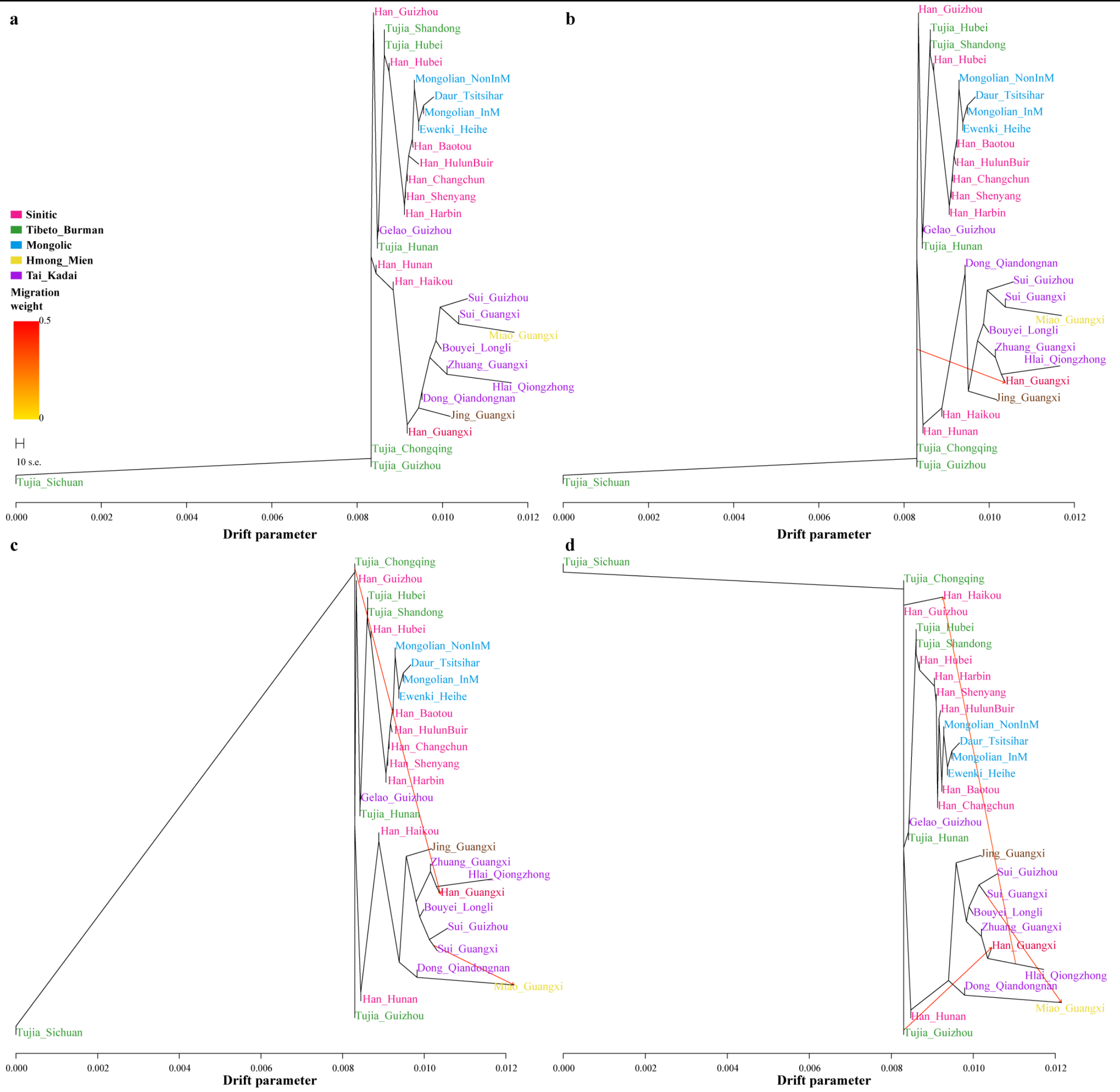

Figure. S10. Inferred phylogenetic relationship using TreeMix.

(a-d) Plotted is the topology of the graph inferred by TreeMix for East Asian groups based on the merged HO dataset, allowing gene flow events from 0 to 3. The populations are colored according to their language family (deep pink: Sinitic, green: Tibeto-Burman, blue: Mongolic, yellow: Hmong-Mien, purple: Tai-Kadai), respectively.

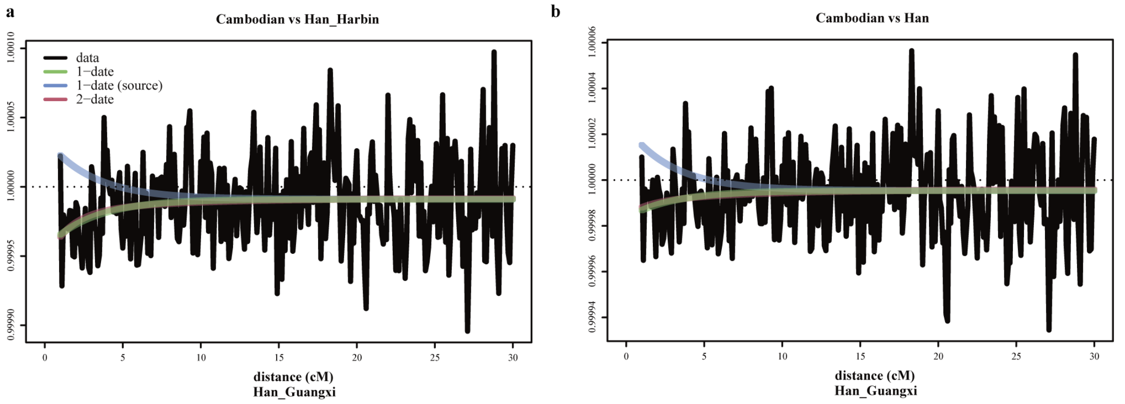

Figure. S11. Overview of inferred admixture for GPH using fastGLOBETROTTER.

(a-b) Results from fastGLOBETROTTER. Original data were displayed as a black line (scaled probability) and the green, red and cyan lines represent the fit of a single date of admixture, two dates of admixture and one date of admixture between two sources, respectively. The admixture conclusion ("one-date", "multiway", "multiple-dates") can be inferred by evaluating which (green, cyan, red) colored lines fit the data (black line) best.

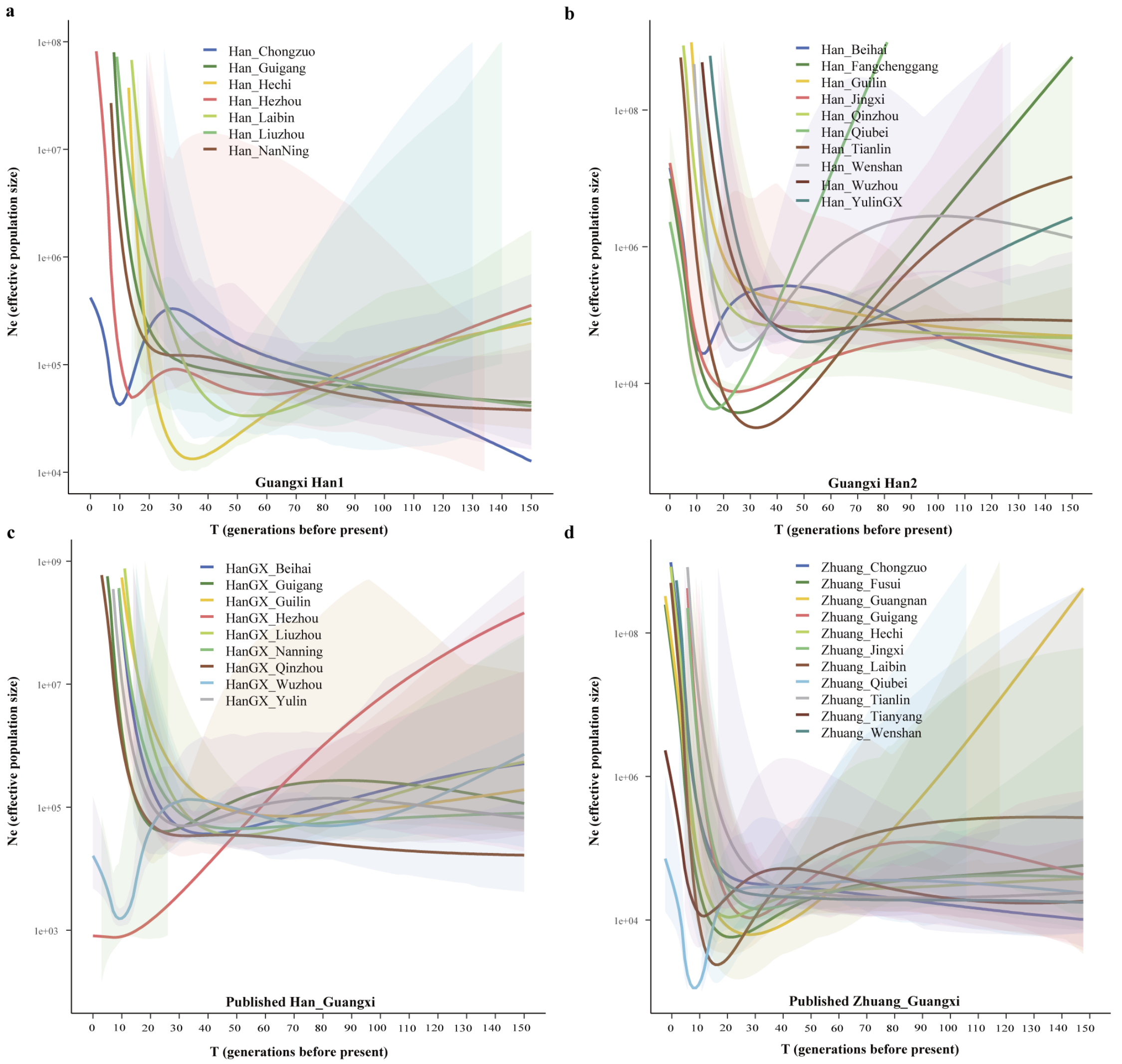

Figure. S12. Effective population sizes of Guangxi groups inferred from IBDNe.
(a-b) The effective population sizes of GPHs. (c) The effective population size of the published GPHs. (d) The effective group size of published Guangxi ethnic minority groups.

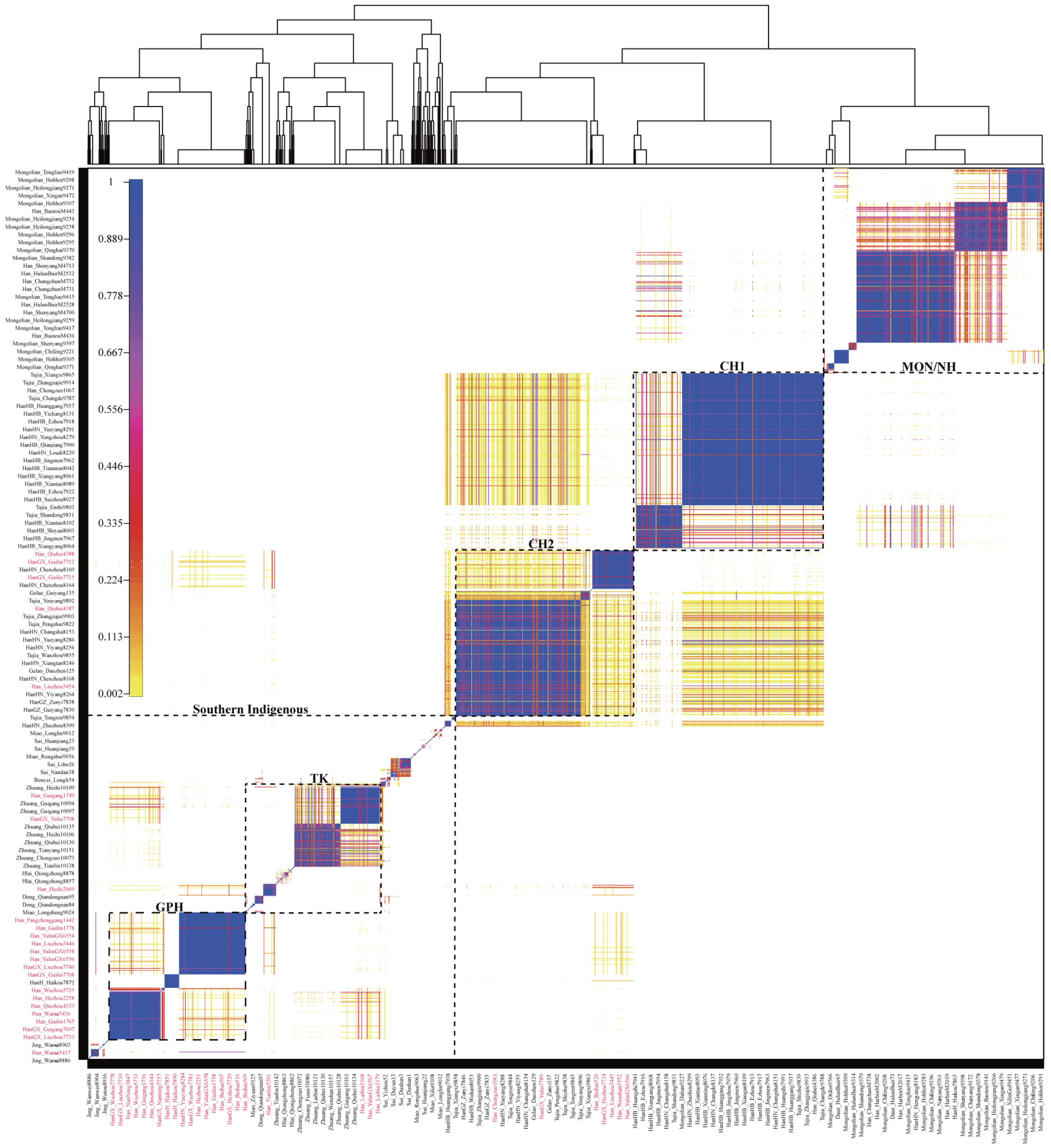

Figure. S13. Fine-scale genetic structure and coancestry curves based on the shared haplotype data.
The pairwise coincidence matrix at the individual level is inferred by fineSTRUCTURE with extremely high (dark blue) and low (yellow) values. There are abbreviations used: GPH, Guangxi Pinghua Han; TK, Tai-Kadai; CH1, Central Han1; CH2, Central Han2; MON, Mongolia; NH, Northern Han.

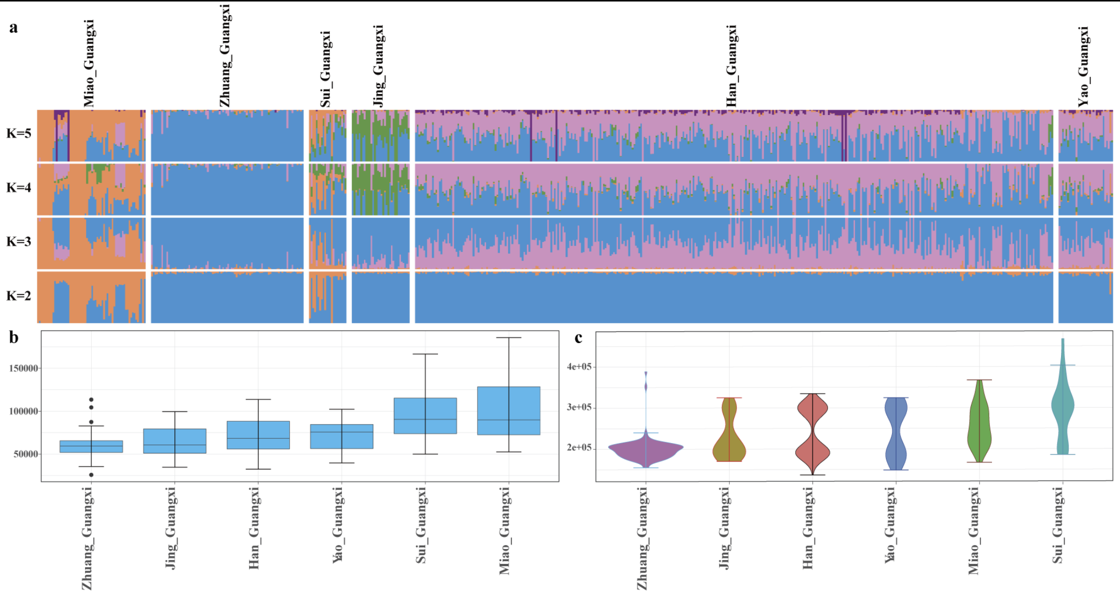

Figure. S14. The population structure of five ethnic communities and GPH in Guangxi Province.
(a) The number of source surrogate populations in ADMIXTURE of 2 to 5, with the lowest cross-validation error at K = 2. (b) Results of ROH analysis (5-15 Mb) reflect a higher probability of consanguineous marriage within the Miao and Sui groups. (c) Comparison of the total number of ROH between groups reflects the different demographic histories.

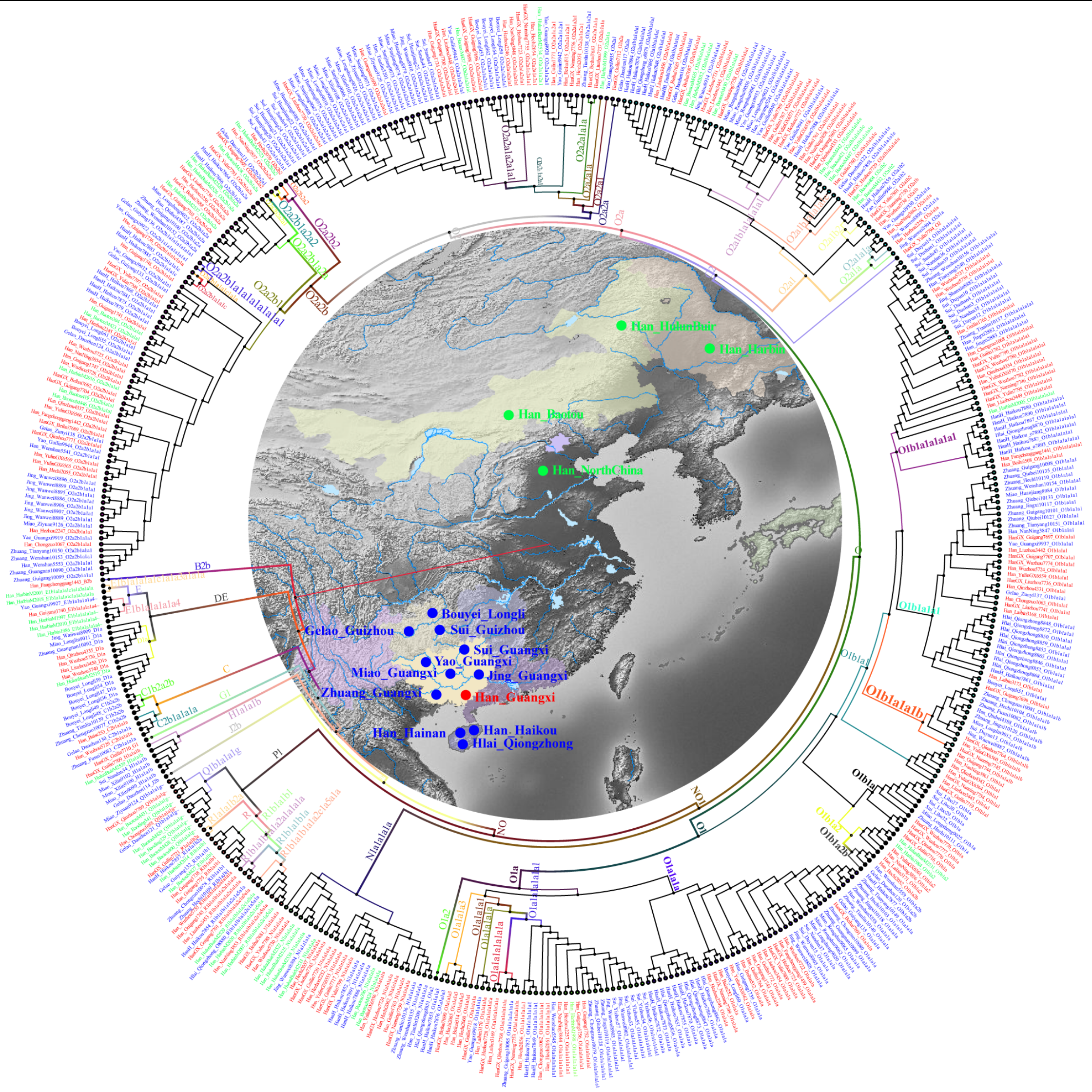

Figure. S15. Paternal lineages in the context of East Asia.

ML tree of 474 chrY sequences. Colors in the different groups correspond to the geographic distribution of the samples (green: Northern Chinese groups, dark blue: Southern Chinese groups, red: GPH). Different haplotypes were colored with different colors randomly.

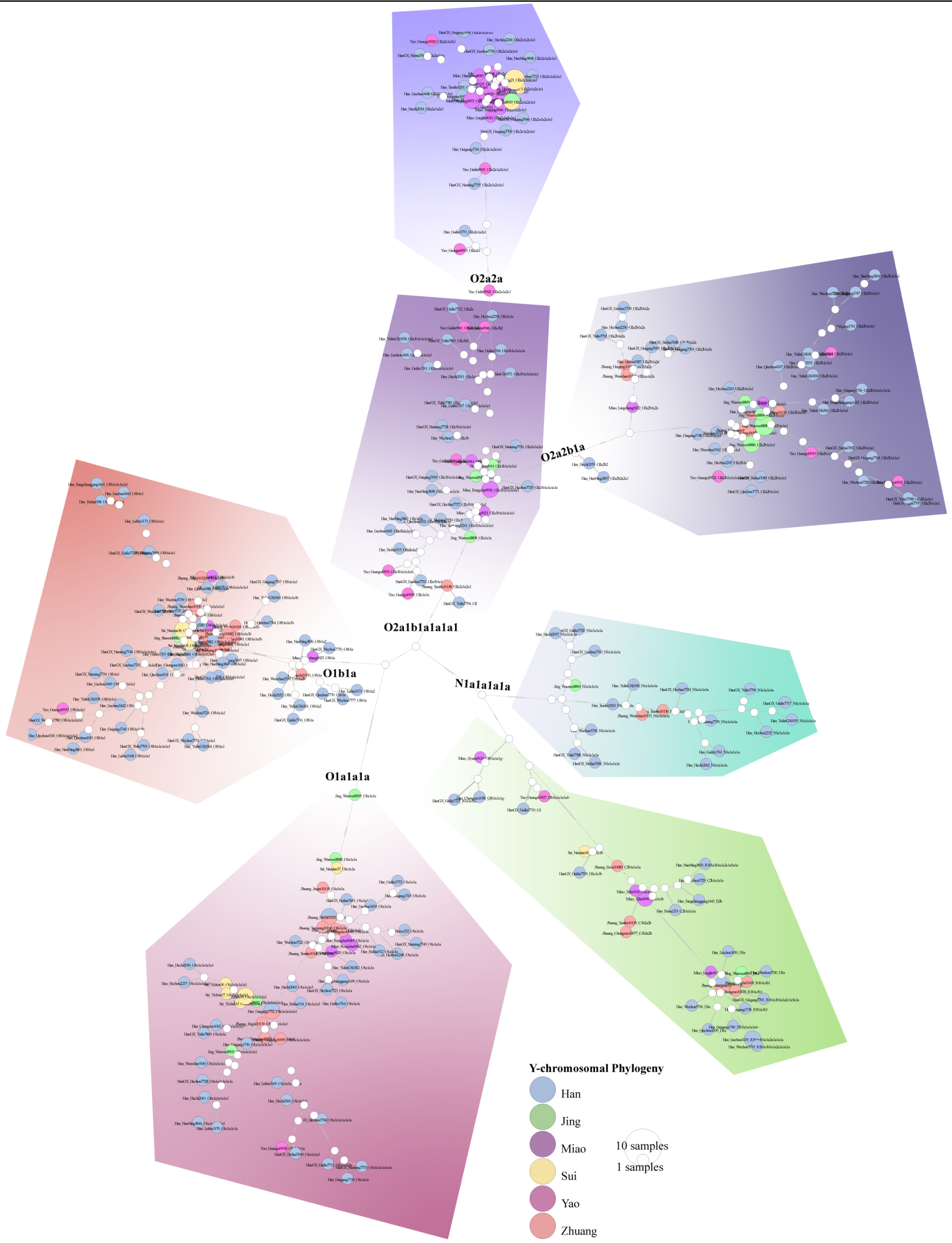

Figure. S16. Y chromosome haplogroup distribution.
Haplotype distribution patterns of 317 individuals from six populations in Guangxi based on Network.

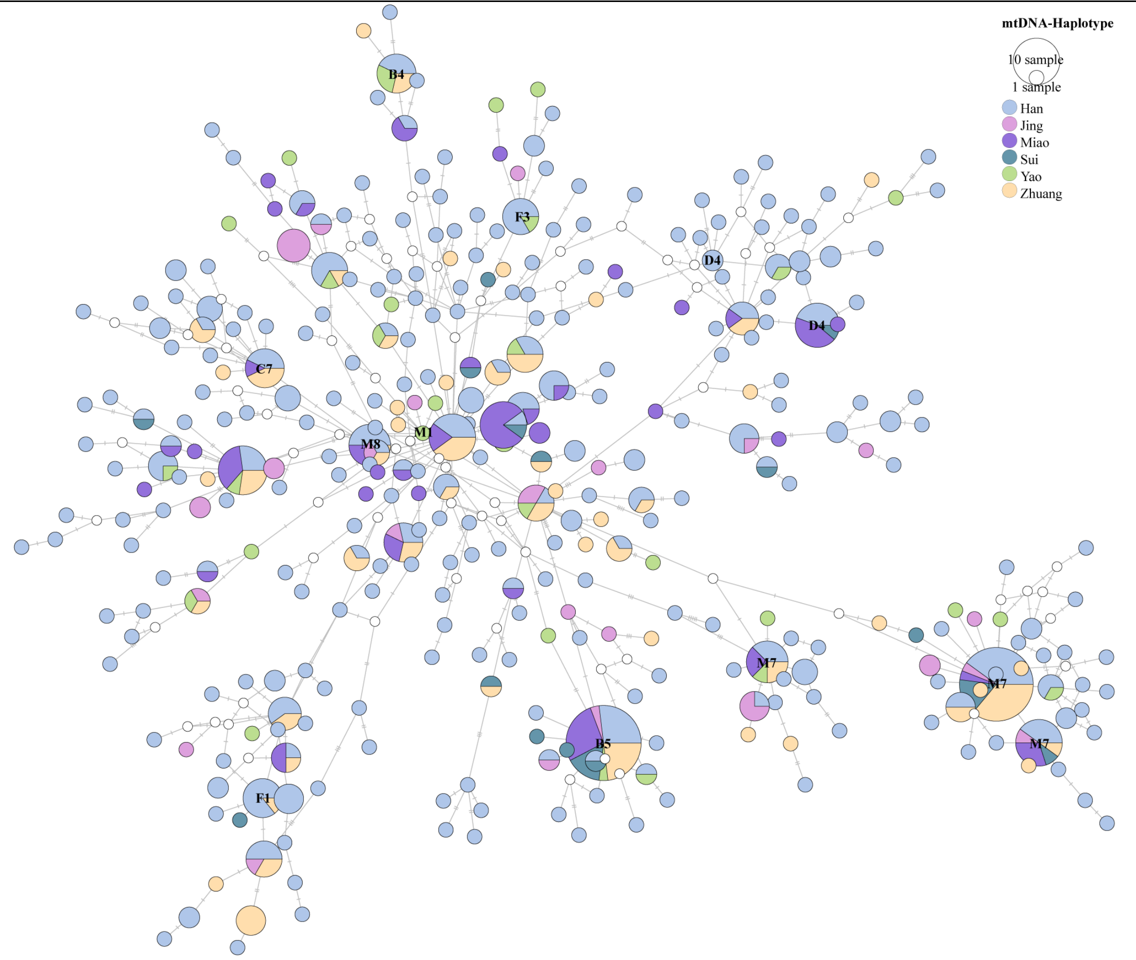

Figure. S17.Mitochondrial haplogroup distribution.

The maternal genetic background of 619 individuals from six groups in Guangxi was revealed using network-based phylogenetic relationships.

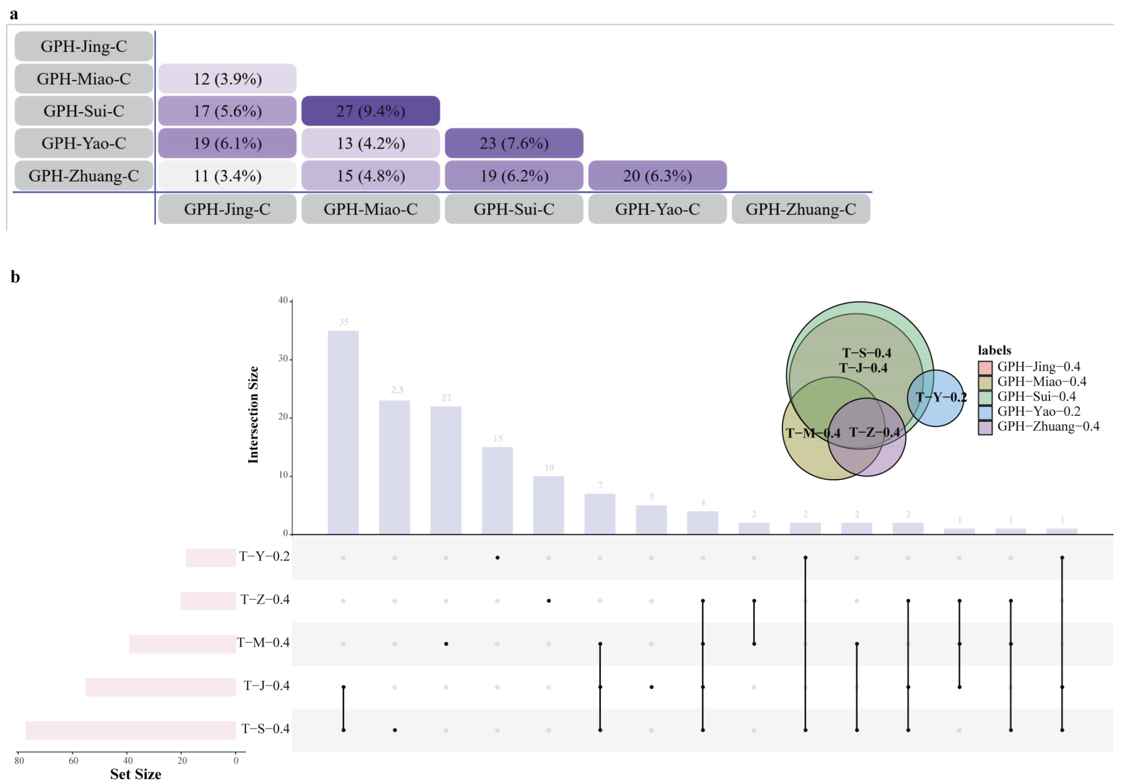

Figure. S18. Natural selection signals and highly differentiated loci.

(a) Venn diagram of regional-specific natural selection signals detected by PBS method (GPH-Jing/Miao/Sui/Yao/Zhuang_Guangxi-Han_Changchun). The number and percentage of intersections of different results are shown. See Additional file 2, Table S12 for more details. (b) Upset plots of highly differentiated loci. Loci with differentiation frequencies higher than 0.4 between populations were selected for comparison (except for the T-Y group, which was 0.2). T, GPH; Y, Yao_Guangxi; Z, Zhuang_Guangxi; M, Miao_Guangxi; J, Jing_Guangxi; S, Sui_Guangxi; C, Han_Changchun.
